# Salivary Exosome Proteomics and Bioinformatics Analysis in 7,12-Dimethylbenz[a]anthracene-Induced Oral Cancer with Radiation Therapy—A Syrian Golden Hamster Model

**DOI:** 10.3390/diagnostics12010065

**Published:** 2021-12-28

**Authors:** Wen-Chen Wang, Ming-Yii Huang, Yuk-Kwan Chen, Wan-Chen Lan, Tzong-Ming Shieh, Yin-Hwa Shih

**Affiliations:** 1 Department of Oral Pathology, College of Dental Medicine, Kaohsiung Medical University, Kaohsiung 80708, Taiwan; wcwang@kmu.edu.tw (W.-C.W.); yukkwa@kmu.edu.tw (Y.-K.C.); 2 Division of Oral Pathology & Maxillofacial Radiology, Kaohsiung Medical University Hospital, Kaohsiung 80708, Taiwan; 3 Oral & Maxillofacial Imaging Center, College of Dental Medicine, Kaohsiung Medical University, Kaohsiung 80708, Taiwan; 4 Department of Radiation Oncology, Kaohsiung Medical University Hospital, Kaohsiung 80708, Taiwan; miyihu@kmu.edu.tw; 5 Department of Radiation Oncology, Faculty of Medicine, College of Medicine, Kaohsiung Medical University, Kaohsiung 80708, Taiwan; 6 School of Dentistry, China Medical University, Taichung 40402, Taiwan; 7 Department of Healthcare Administration, Asia University, Taichung 41354, Taiwan; magic1986713@hotmail.com

**Keywords:** DMBA, OSCC, radiation, salivary exosome, proteomics

## Abstract

Exosomes carry cellular proteins and contain molecules that can be potential biomarkers of diseases. This study used a Syrian golden hamster model of 7,12-dimethylbenz[a]anthracene (DMBA)-induced oral squamous cell carcinoma with radiation therapy to exclude the confounding factors that may affect outcomes in clinical studies, and re-examine the role of exosomes during tumorigenesis. We used data-dependent acquisition-based quantitative proteomics and bioinformatics analyses and found unique proteins present (desmocollin-2) or absent (Glucagon-cAMP-PKA-CREB pathway-related proteins) in the salivary exosomes of the pre-radiation DMBA-treated group (PreD). Comparing our data to other studies, salivary exosomes in the PreD group were found carrying proteins that the tumor mass does not express and lacking the proteins needed during tumorigenesis. Immunohistochemistry staining showed p53 expression but a negative apoptotic signal in the PreD tumor tissue. We thus suggest that inhibition of desmocollin-2 expression in tumor tissue may impede the activation of cell apoptosis. However, both the origin of the salivary exosomes and main role of the salivary exosome proteins should be clarified in future studies.

## 1. Introduction

Exosomes are extracellular nanovesicles with sizes between 30–150 nm secreted by cells carrying biomolecules and which play a role in physiological and pathological processes [1,2]. Exosomes exist in body fluids including blood, urine, saliva, breast milk, cerebrospinal fluid, and peritoneal fluid [3]. The molecules of salivary exosomes are similar to those in the exosomes of different origins, such as urinary exosomes [4] and serum exosomes [5]. Thus, salivary exosome proteins reflect the microenvironmental changes around local lesions as well as the systematic conditions during tumorigenesis [6].

Over the past decade, several exosomal molecules (DNA, RNA, and proteins) [7,8,9,10] have been established as cancer biomarkers. Some of the exosomal markers identified in cancer specimens support the idea that cancer cells release exosomes around their microenvironment and promote cancer progression [11].

DMBA is an immunosuppressor and carcinogen that is widely used in cancer research as a tumor initiator [12]. DMBA-induced oral squamous cell carcinoma (OSCC) in the Syrian golden hamster (*Mesocricetus auratus*) is a well-established and widely used model in OSCC research [13] because the tumorigenesis process is similar between humans and hamsters [14].

Investigation of exosomal biomarkers is a leading trend in cancer research. However, carcinogenesis may be initiated through different mechanisms and may be influenced by the diversity of species and environments. This study minimized the confounding factors that may affect gene expression by using the DMBA-induced OSCC Syrian golden hamster (*Mesocricetus auratus*) model along with radiation treatment. We further analyzed the proteomics results from the bioinformatics databases. The outcomes of this study provide clues regarding the role of exosomes in tumorigenesis.

## 2. Materials and Methods

### 2.1. DMBA-Induced Tumorigenesis

Outbred, young (6-week-old), male Syrian golden hamsters (*Mesocricetus auratus*; n = 42, purchased from the National Laboratory Animal Center, Taipei, Taiwan), weighing approximately 100 g at the beginning of the experiment, were randomly divided into DMBA and control groups; each group had 3 animals. The animals were housed under constant conditions (22 °C, 12-h light/dark cycle) and were supplied with tap water and standard Purina laboratory chow ad libitum.

After allowing the animals to acclimatize to their new surroundings, both pouches of the animals were painted with 0.5% DMBA solution (wt/vol) in mineral oil using a No. 4 sable-hair brush, at 9 a.m. every Monday, Wednesday, and Friday, for 12 weeks. The pouches of control hamsters were painted with mineral oil.

### 2.2. Radiation Treatment Procedure of the Local Tumor Mass

The radiation protocol basically followed our previous study [15]. The animals were placed in custom-made acrylic containers constructed to expose only the head; the remaining parts of the animals were protected using a lead shield. Subsequently, only the heads received fractionated radiation, with a total radiation dose of 42 Gy (6 MV, 7 Gy/twice/week) using a linear accelerator (Varian 2100C, Palo Alto, CA, USA).

### 2.3. Immunohistochemistry Protocol

The pouch tissues were dissected and immediately fixed with formaldehyde solution for 24 h. Specimen slides were prepared using 4-µm-thick sections by the Division of Oral Pathology & Maxillofacial Radiology, Kaohsiung Medical University Hospital. The slides were deparaffinized using xylene and ethanol. Antigen retrieval was conducted using an antigen retrieval buffer (Tris/EDTA, sodium citrate) in a pressure cooker for 3 min. Antigen detection was performed using the Novolink polymer detection system (Leica Biosystems, Harbourfront Centre, Singapore) according to the manufacturer’s protocol. The p53 monoclonal antibody was diluted 1:50 (Novocastro, Newcastle, UK). TUNEL assay was conducted using an in situ cell death detection kit (Roche, Basel, Switzerland).

### 2.4. Saliva Collection

The hamsters were anesthetized using 50 mg/mL Zoletil 50^®®^ (Virbac, Carros, France) via peritoneal injection. When the hamsters were in a state of delirium, we injected pilocarpine HCl Oph. A solution (2 mg/mL) (Alcon, Fort Worth, TX, USA) into the peritoneum at 1 mL/kg to stimulate saliva secretion. The hamsters were laid at a 45° angle with the head down, and the total saliva was aspirated into a microcentrifuge using a pipette. After 25 min of collection, the hamsters were kept in a warm observation chamber for recovery.

### 2.5. Saliva Exosome Isolation and Protein Extraction

Salivary exosomes were isolated by ultracentrifugation. The hamster saliva was centrifuged at 900× *g* for 15 min to remove debris. The supernatant was sequentially filtrated through a 0.45 μm filter at 14,000× *g* for 40 min with a 100 K microsep advance centrifugal device (PALL Life Science, New York, NY, USA) at 14,000× *g* for 40 min. The flow-through was collected and ultra-centrifuged using the Optima L-90K system at 200,000× *g* at 4 °C for 16 h with an SW41 Ti rotor (Beckman Coulter, CA, USA). The exosome pellet at the bottom of the ultracentrifuge tube was then lysed with 20 μL RIPA buffer (Millipore, MA, USA). The samples were frozen overnight at −20 °C and centrifuged at 13,800× *g* for 20 min. The supernatant was collected, and the protein concentration was determined using the Bio-Rad protein assay reagent (Bio-Rad Laboratories, Hercules, CA, USA). Protein samples (5 μg) were separated by SDS-PAGE until a straight-line pattern was observed in the stacking gel. The gel was then stained with 0.5% Coomassie blue. The bands on the gel were cut out, transferred to a 1.5 mL centrifuge tube, and stored at 4 °C for further proteomic analyses. The CD63 biomarkers of the salivary exosomes were detected (Supplementary Figure S1) using CD63 mouse monoclonal antibody (Santa Cruz Biotechnology, TX, USA).

### 2.6. In-Gel Digestion and LC-MS/MS Analysis

The excised gel bands were cut into small pieces and washed three times with 25 mM ammonium bicarbonate (ABC, pH 8.2) containing 50% ACN for 15 min. The gel pieces were dehydrated with 100% acetonitrile and digested with trypsin (1:50 trypsin to protein ratio in weight) in 25 mM ABC at 37 °C overnight. After digestion, the tryptic peptides were extracted from the gel using 0.1% TFA in 50% acetonitrile. All extracted solutions were combined and concentrated using a centrifugal concentrator. The dried samples were stored at −20 °C.

Samples were resuspended in 0.1% formic acid (FA) and analyzed using an Ultimate 3000 RSLCnano system (Thermo Fisher Scientific) coupled with an Orbitrap Exploris 480 mass spectrometer (Thermo Fisher Scientific). Samples (3 µL) were injected onto an EASY-Spray^TM^ PepMap^TM^ RSLC C18 column (25 cm × 75 μm ID, 2 μm particle size, 100 Å pore size; P/N ES902; Thermo Scientific, Waltham, MA, USA) at a flow rate of 300 nL/min. The nanoLC gradient conditions were as follows: 2% to 32% (*v*/*v*) buffer B (80%ACN/0.1% FA) for 68 min, 32% to 55% B for 5 min, 32% to 90% B for 6 min, and then returned to 98% buffer A (0.1% FA) for 11 min. The Orbitrap Exploris 480 mass spectrometer was operated in the positive ion mode. The electrospray voltage was set at 1900 V and the temperature of the ion transfer tube was set at 275 °C. Data-dependent acquisition (DDA) parameters were set as follows: For MS1, the resolution was set at 120,000. The scan range was 375–1500 m/z. The normalized automatic gain control (AGC) target was 300%, and the charge state was 2–6. The intensity threshold was set at 5.0 × 10^4^. The dynamic exclusion time was set at 20 s, with a mass tolerance of 10 ppm. The cycle time was set at 3 s. For MS2, the resolution was set at 30,000. The normalized AGC target was set at 200%. The normalized HCD collision energy was set at 30%.

### 2.7. Data Quantification and Statistical Analysis

Raw data were imported into Proteome Discoverer 2.4 software. For protein identification, the SEQUEST node was set up to search data against the SwissProt database (*Mesocricetus auratus*, release 2021_09; 32,294 sequences, 20,404,560 residues). The search parameters for precursor ion and fragment ion tolerance were 10 ppm and 0.02 Da, respectively. The enzyme specificity was set as trypsin, and the maximum missed cleavage was set at 2. The fixed modification was set as carbamidomethyl (C), and variable modifications were set as oxidation (M) and deamidation (NQ). The false discovery rate (FDR) was estimated to be <1%. In label-free quantification, the precursor ion intensity was estimated as the relative abundance of the identified protein. The protein abundance in each sample was normalized to the sum of all peptide amounts for a given protein. Statistical analysis was performed using *t*-test, and statistical significance was set at *p*-value below 0.05.

### 2.8. Bioinformatics Analysis

We conducted gene ontology analysis and Reactome pathway analysis using PANTHER (Protein Analysis through Evolutionary Relationships) database version 16.0 (http://www.pantherdb.org) (5 November 2021) [16]. This database includes a library of over 15,000 phylogenetic trees, and the functional classifications include gene ontology terms and pathways. We then analyzed the biological processes, molecular functions, and cellular components. All results shown are valid for an overall false discovery rate (FDR) <0.05, and raw *p*-value < 0.05, as determined by the Benjamini–Hochberg test and Fisher’s exact test, respectively. Fold enrichment indicates the gene expression observed in the uploaded list over the expected list. If this was greater than 1, this category was considered as overrepresented in our experiment. Conversely, the category was considered as underrepresented if it was less than one.

## 3. Results

### 3.1. The Pouches Showed a Tumor Mass after 12 Weeks of DMBA Treatment and the Tumor Mass Showed Apoptosis after Receiving Six Rounds of Radiation Exposure

**Figure 1:**
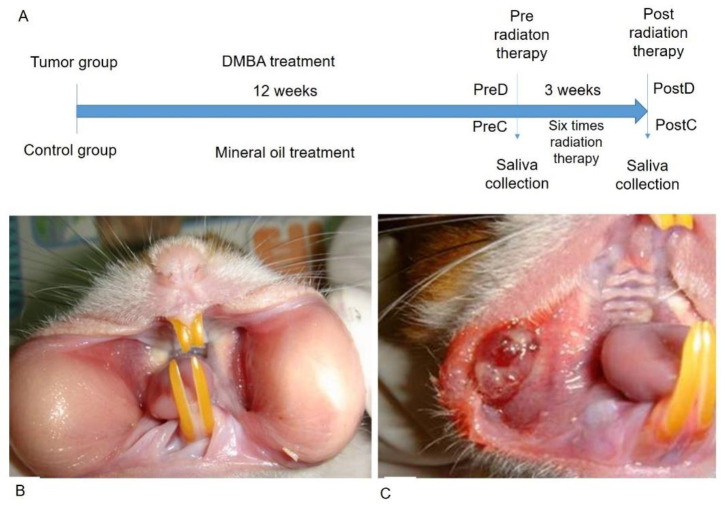
DMBA-induced tumor mass in the buccal pouch of the golden hamster. (**A**): Time frame of the research design; (**B**): Normal buccal pouch; (**C**): DMBA-induced tumor in buccal pouch.

**Figure 2:**
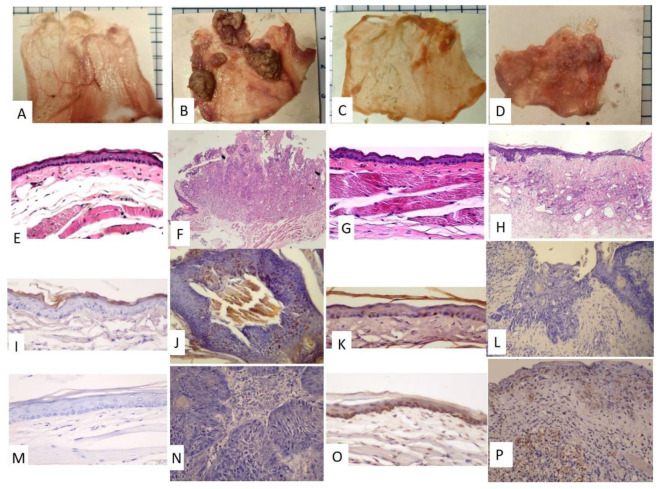
Gross examination of the pouch specimens, HE staining, p53 staining, and TUNEL staining. (**A**–**D**) Pouch specimens (scale bar: 5 mm –the blue line spacing on the margin of the photo) (**A**,**E**,**I**,**M**): PreC; (**B**,**F**,**J**,**N**): PreD; (**C**,**G**,**K**,**O**): PostC; (**D**,**H**,**L**,**P**): PostD. (**E**–**H**), HE staining, (**E**,**G**): 100×; (**F**,**H**): 40×. (**I**–**L**), p53 staining, 200×, (**I**,**L**): negative; (**J**,**K**): partially positive (**M**–**P)**, TUNEL staining, 200×. (**M**,**N**): negative, (**O**,**P**): positive.

The time frame of the research design is shown in Figure 1A. The golden hamsters were randomly separated into control and DMBA treatment groups. The DMBA group was brushed with DMBA, and the control group was brushed with mineral oil on the buccal pouch three times a week. After 12 weeks, the DMBA-treated golden hamsters showed a tumor mass on the buccal pouch (Figure 1C) compared to the control group (Figure 1B). We also collected the pre-radiation therapy saliva of both groups. The control group was labeled as PreC, and the tumor group was labeled as PreD. We administered radiation therapy twice per week for 3 weeks to both groups. The post-radiation saliva was collected; the saliva of the control group was labeled as PostC, and the tumor group was labeled as PostD. Both pre- and post-radiation salivary exosomes were isolated by ultracentrifugation, and exosomal protein was extracted for differential protein expression analysis. The golden hamsters were then sacrificed and pouch specimens were subjected to gross examination, HE staining, p53 staining, and TUNEL staining (Figure 2). HE staining showed that the tumor masses of the PreD and PostD groups were poorly differentiated (Figure 2F,H) and the PostD tissue showed shrinkage and apoptosis (TUNEL positive) after six rounds of radiation (Figure 2D,P).

### 3.2. The DMBA-Treated PreD Group Expressed a Unique Salivary Exosome Protein, Desmocollin-2

The differentially expressed proteins were selected based on two features including uniquely expressed protein in one group and *p*-value < 0.05 (Student’s *t*-test). In total, exosomal proteins were quantified using a data-dependent acquisition method. Among the four groups, the unique proteins in the PreD salivary exosomes were desmocollin-2 isoforms X1, X2, and X3 (Table 1). Desmocollin-2 is a cadherin-type protein that links the adjacent cells in desmosomes. Based on the literature review, DMBA-induced malignant transformation of oral mucosa epithelium reduced the number of desmosomes, and the Desmocollin-2 expression level was reduced in head and neck cancer tissues with poor clinical outcomes. Our data revealed that salivary exosomes of the PreD group carry proteins that are absent in the tumor tissue during tumorigenesis.

### 3.3. Five Proteins Related to Biomolecular Breakdown Were Absent in Salivary Exosomes in the PostD Group

Comparing the four groups, five unique differentially expressed proteins were absent in the PostD group: serpin A9 (serping9), peroxisomal multifunctional enzyme type 2 (Hsd17b4), alpha-mannosidase OS (Man2b2), heat shock 70 kDa protein 13 (Hspa13), and dipeptidyl peptidase 2 (DPP7). The detailed protein functions are listed in Table 1. Our data revealed that some enzyme functions correlated with protein digestion, lipid oxidation, glycoprotein turnover, and oligopeptide degradation were lacking in the salivary exosomes in the PreC and PostD groups. We suggest that these proteins are associated with cellular recovery in normal tissues and post-tumor exposure to radiation treatment.

### 3.4. Salivary Exosomes of PreD Lacked the Proteins Associated with Tumorigenesis Signaling Pathways and the p53-Dependent and -Independent DNA Repair Pathway

**Figure 3:**
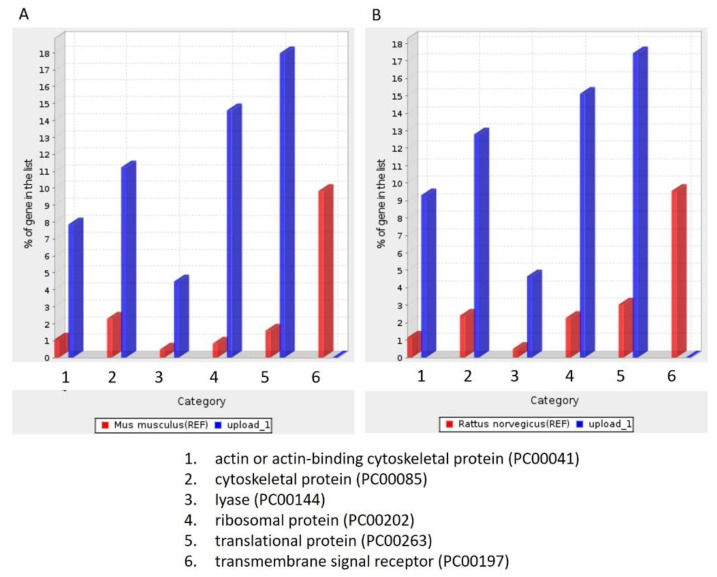
PANTHER protein class analysis. (**A**): Based on the *Mus musculus* database; (**B**): Based on *Rattus norvegicus* database.

To identify the unique proteins involved in tumorigenesis during DMBA treatment, we compared the differentially expressed proteins among the four groups (PreD, PreC, PostD, and PostC) and identified the proteins absent in the PreD group. One hundred and seventy-four proteins were identified and are listed in Supplementary Table S1. To further understand the roles of these 174 differentially expressed proteins, we queried PANTHER database version 16 and annotated the GO terms based on PANTHER protein class (Figure 3), Reactome pathway (Table 2), PANTHER GO-Slim Molecular Function (Table 3), PANTHER GO-Slim Biological Process (Table 4), and PANTHER GO-Slim Cellular Component analysis (Table 5). However, the website does not provide a database for Syrian golden hamsters (*Mesocricetus auratus*). We thus used both rat (*Rattus norvegicus*) and mouse (*Mus musculus*) databases as references. The outcomes showed six main classes of these 174 proteins, including actin-or actin-binding cytoskeletal proteins, cytoskeletal proteins, lyase, ribosomal proteins, transitional proteins, and transmembrane signal receptors. These proteins function as signal reception and cytoskeletal structures (Figure 3). We obtained 136 Reactome pathways in the *Rattus norvegicus* database and 132 Reactome pathways in the *Mus musculus* database (Supplementary File S1). Among them, CREB1 phosphorylation through the activation of adenylate cyclase, PKA activation in glucagon signaling, and glucagon signaling in metabolic regulation were the three leading pathways (fold enrichment > 70%) in the Reactome analysis. To our knowledge, cAMP regulates cell transcription through protein kinase A (PKA) and its downstream effector, cAMP-responsive element binding protein (CREB). The cAMP–PKA–CREB signaling pathway is associated with tumor growth, migration, and glucose homeostasis. Glucagon signaling promotes glycogenolysis and gluconeogenesis through the cAMP-PKA signaling pathway, and glycogenolysis is enhanced in the oral dysplastic/malignant epithelium. Our data indicated the absence of proteins associated with oral tumorigenesis in salivary exosomes after 12 weeks of DMBA treatment. We also found that these proteins were implicated in the p53-dependent and -independent DNA damage checkpoint and response pathways. As the fold enrichment was low (11 to 15), this category had a minor impact on tumorigenesis after 12 weeks of DMBA treatment. In summary, after 12 weeks of DMBA treatment, a tumor was induced in the buccal pouches. The proteins associated with tumorigenesis were lacking in salivary exosomes. In contrast, proteins lacking in the tumor tissue were expressed in salivary exosomes. Our data revealed that the expression of salivary exosome proteins contrasted that of proteins expressed in buccal tumor cells.

### 3.5. Molecular Function, Biological Process, and Cellular Component Analysis

The classification results of the molecular function are listed in Table 3. Fourteen molecular function ontologies were found in the *Rattus norvegicus* database and 17 were found in the *Mus musculus* database. The three leading functions in the *Rattus norvegicus* database were signal sequence binding (GO:0005048), ubiquitin-like protein conjugating enzyme activity (GO:0061650), and actin filament binding (GO:0051015). The three leading functions in the *Mus musculus* database were signal sequence binding (GO:0005048), structural constituent of ribosome (GO:0003735), and actin filament binding (GO:0051015). The data revealed that the proteins lacking in the salivary exosomes of the PreD group were associated with molecular binding in cells. The classification results of the biological processes are listed in Table 4. Twenty-two biological functions were found in the *Rattus norvegicus* database and twenty-three were listed in the *Mus musculus* database. According to the fold enrichment, protein K48-linked ubiquitination (GO:0070936) and regulation of endocytosis (GO:0030100) were more than 10-fold enriched in the *Rattus norvegicus* database, and regulation of endocytosis (GO:0030100) was more than 10-fold enriched in the *Mus musculus* database. The data revealed that the proteins lacking in the PreD group were mainly responsible for endocytosis and 53BP1 recruitment at the DNA damage site. The classification results of the cellular components are listed in Table 5. Twenty-nine proteins were found in the *Rattus norvegicus* database and thirty-two were listed in the *Mus musculus* database. The main three leading classes were related to the proteasome in both the *Rattus norvegicus* and *Mus musculus* databases. Upon comparison with Figure 2, p53 was found to be positive in PreD tissues and provided evidence that the DNA damage repair system was activated during tumorigenesis.

## 4. Discussion

This study used a hamster model to reduce the confounding factors introduced by differences in species and environments and to explore the unique proteins associated with DMBA-induced tumorigenesis in the buccal pouch. Buccal pouch necropsy showed that the tumor mass arose after DMBA treatment and shrank after radiation. HE staining showed poor morphology on the tumor mass. TUNEL staining showed that apoptosis was induced upon radiation treatment. We found one unique salivary exosome protein, desmocollin-2, present in the PreD group that was absent in the DMBA-induced tumor tissue, as reported in other studies [17,18]. Five proteins were not expressed in the salivary exosomes of the PostD group, and these were responsible for peptide and fatty acid metabolism. The unique salivary exosome proteins that were absent in the PreD group were associated with the tumorigenesis signaling pathway and DNA damage repair systems.

We found that p53 was expressed in the PreD and PostC groups (Figure 2), and that the TUNEL signal (apoptosis marker) was only detected in the PostC and PostD groups (Figure 2). This revealed that p53-dependent cell apoptosis was defective in the PreD group (p53 positive and TUNEL negative). The radiation treatment activated apoptosis in PostC and PostD, and shrank the tumor mass in PostD (Figure 2, TUNEL positive). The data revealed that the apoptosis mechanism was blocked by DMBA treatment. We further compared the salivary exosome protein expression and identified candidate proteins that may impede apoptosis during DMBA treatment. We found a unique protein, desmocollin-2, in the PreD group. Desmosomes are made up of desmoglein-2 and desmocollin-2, which are affiliated with the underlying intermediate filaments via linker proteins to provide mechanical strength to epithelia. Both desmoglein-2 and desmocollin-2 are cleaved after apoptosis onset [19]. Desmoglein-2 is a novel regulator of apoptosis. Downregulation of Dsg2 in the intestinal epithelium protects cells from apoptosis [20]. To our knowledge, no evidence has shown an association between desmocollin-2 expression and apoptosis in cancer cells, and numerous studies have shown a lack of desmocollin-2 expression in oral tumor tissues [18,21]. Further studies are needed to clarify the association between desmocollin-2 expression and tumor cell apoptosis.

We found five unique proteins lacking salivary exosomes in the PreC and PostD groups (Table 1). Based on these data, we suggest that the oral microenvironment returns to a normal status after radiation treatment. Four of the proteins are associated with peptide and lipid metabolism, which are involved in anti-tumor mechanisms [22,23,24,25]. We also found 174 unique proteins that were absent in the PreD group (Supplementary Table S1) which are associated with tumorigenesis signaling (Table 2), based on the unique markers present and absent in the PreD group. We hypothesized that salivary exosomes act as recycling bins that pack proteins not expressed by cancer cells. Whether exosomes are secreted from cells surrounding the tumor mass to rescue the damage site or by the tumor mass itself needs to be clarified in future studies.

If the recycling bin hypothesis for exosomes is valid, based on the exosomal proteins absent in the PreD group, we suggest that the PreD group showed tumorigenesis through the Glucagon-cAMP-PKA-CREB1 signaling pathway (Table 2), consistent with previous findings, in which an antidiabetic drug and PKA inhibitor could inhibit DMBA-induced tumorigenesis [26,27]. Further, based on GO analysis, either a p53-dependent or -independent DNA damage response system was activated in the PreD group, and IHC data indicated that p53 positivity supported our hypothesis (Figure 2J).

This study had some limitations. We used the Syrian golden hamster as a model to detect salivary biomarkers, which cannot represent the actual physiological reaction of *Homo sapiens*. We stimulated saliva secretion using pilocarpine, whose overdose may cause trouble breathing. We tried our best to use an effective and safe dose of pilocarpine to collect 1 mL of saliva per hamster. We finally obtained a total of 5–10 μg of exosomal proteins from the saliva of each animal. Proteomics analysis required 5 μg of exosomal proteins and made it impossible for us to verify the expression of other exosomal proteins. Therefore, further studies are needed to identify the salivary exosome proteins as in this study, by increasing the number of animals as needed.

## 5. Conclusions

We found that salivary exosomes carry proteins absent in tumor tissue and lack proteins that should be expressed in tumor tissue. The origin of salivary exosomes and the main role of salivary exosome proteins should be clarified in future studies.

## Figures and Tables

**Table 1:** Protein expression values in salivary exosomes pre and post radiation treatment.

		Pre-Radiation		Post-Radiation		
Protein Name	Gene Name	Control	Tumor	Control	Tumor	Protein Function
desmocollin-2 isoform X2	Dsc2	UD	400	UD	UD	Component of intercellular desmosome junctions Contribute to epidermal cell positioning (stratification).
desmocollin-2 isoform X1	Dsc2	UD	400	UD	UD	
desmocollin-2 isoform X3	Dsc2	UD	400	UD	UD	
serpin A9	Serpina9	UD	108.2	291.8	UD	Protease inhibitor that inhibits trypsin and trypsin-like serine proteases.
peroxisomal multifunctional enzyme type 2	Hsd17b4	UD	39.9	360.1	UD	Bifunctional enzyme acting on the peroxisomal beta-oxidation pathway for fatty acids.
alpha-mannosidase	Man2b2	UD	295.9	104.1	UD	Necessary for the catabolism of N-linked carbohydrates released during glycoprotein turnover.
heat shock 70 kDa protein 13	Hspa13	UD	333.4	66.6	UD	Plays a pivotal role in the protein quality control system, ensuring the correct folding of proteins, re-folding of misfolded proteins and controlling, and targeting of proteins for subsequent degradation
dipeptidyl peptidase 2	Dpp7	UD	331.5	68.5	UD	Plays an important role in the degradation of some oligopeptides

The protein values were quantified by data-dependent acquisition. UD: undetectable.

**Table 2:** The tumorigenesis-associated Reactome pathways of the differentially expressed proteins that were absent in the PreD group.

Reference	Reactome Pathways	Fold Enrichment	Raw *p*-Value	FDR
*Rattus norvegicus*	CREB1 phosphorylation through the activation of adenylate cyclase (R-RNO-442720)	>100	3.23 × 10^−4^	1.20 × 10^−2^
	PKA activation in glucagon signalling (R-RNO-164378)	>100	3.23 × 10^−4^	1.18 × 10^−2^
	Glucagon signaling in metabolic regulation (R-RNO-163359)	71.71	5.50 × 10^−4^	1.74 × 10^−2^
	p53-independent G1/S DNA damage checkpoint (R-RNO-69613)	15.06	1.22 × 10^−^^3^	2.87 × 10^−2^
	p53-independent DNA damage response (R-RNO-69610)	15.06	1.22 × 10^−^^3^	2.83 × 10^−2^
	p53-dependent G1/S DNA damage checkpoint (R-RNO-69580)	11.95	2.31 × 10^−^^3^	3.64 × 10^−2^
	p53-dependent G1 DNA damage response (R-RNO-69563)	11.95	2.31 × 10^−^^3^	3.60 × 10^−2^
*Mus musculus*	CREB1 phosphorylation through the activation of adenylate cyclase (R-MMU-442720)	98.82	3.33 × 10^−4^	1.25 × 10^−2^
	PKA activation in glucagon signalling (R-MMU-164378)	98.82	3.33 × 10^−4^	1.22 × 10^−2^
	Glucagon signaling in metabolic regulation (R-MMU-163359)	70.59	5.68 × 10^−4^	1.87 × 10^−2^
	p53-independent G1/S DNA damage checkpoint (R-MMU-69613)	14.53	1.35 × 10^−3^	3.10 × 10^−2^
	p53-independent DNA damage response (R-MMU-69610)	14.53	1.35 × 10^−3^	3.06 × 10^−2^
	p53-dependent G1/S DNA damage checkpoint (R-MMU-69580)	11.58	2.52 × 10^−3^	3.95 × 10^−2^
	p53-dependent G1 DNA damage response (R-MMU-69563)	11.58	2.52 × 10^−3^	3.92 × 10^−2^

Fold enrichment: The category was overrepresented in our experiment if it was greater than 1. Raw *p*-values were determined by Fisher’s exact test. This was the probability that the number of genes we entered into the database in this category occurred by chance (randomly), as determined by the reference list. The closer the *p*-value was to zero, the more significant was the GO term associated with the group of genes. The false discovery rate was calculated using the Benjamini–Hochberg procedure. By default, a critical value of 0.05 was used to filter the results. All results shown are valid with an overall FDR < 0.05.

**Table 3:** PANTHER GO-Slim molecular function analysis.

Reference List		Reference List	
*Rattus norvegicus*	Fold Enrichment	*Mus musculus*	Fold Enrichment
signal sequence binding (GO:0005048)	23.53	signal sequence binding (GO:0005048)	24.71
ubiquitin-like protein conjugating enzyme activity (GO:0061650)	17.51	structural constituent of ribosome (GO:0003735)	22.06
actin filament binding (GO:0051015)	12.45	actin filament binding (GO:0051015)	12.56
lyase activity (GO:0016829)	8.88	structural molecule activity (GO:0005198)	10.47
actin binding (GO:0003779)	8.66	lyase activity (GO:0016829)	9.24
structural constituent of ribosome (GO:0003735)	7.21	actin binding (GO:0003779)	8.77
mRNA binding (GO:0003729)	6.34	molecular adaptor activity (GO:0060090)	8.75
RNA binding (GO:0003723)	5.81	mRNA binding (GO:0003729)	8.72
GTPase activity (GO:0003924)	5.4	RNA binding (GO:0003723)	7.72
structural molecule activity (GO:0005198)	5.27	GTPase activity (GO:0003924)	5.43
protein-containing complex binding (GO:0044877)	5.24	protein-containing complex binding (GO:0044877)	5.15
cytoskeletal protein binding (GO:0008092)	4.64	cytoskeletal protein binding (GO:0008092)	4.61
binding (GO:0005488)	1.61	nucleic acid binding (GO:0003676)	2.18
molecular_function (GO:0003674)	1.47	heterocyclic compound binding (GO:1901363)	2.05
		organic cyclic compound binding (GO:0097159)	2.01
		binding (GO:0005488)	1.67
		molecular_function (GO:0003674)	1.5

All results shown are valid for an overall FDR < 0.05, and a raw *p*-value < 0.05, as determined by Fisher’s exact test. Fold enrichment is the gene expression observed in the uploaded list over that in the expected list. If it was greater than 1, the category is overrepresented in our experiment. Conversely, the category is underrepresented if it is less than 1. GO: Gene ontology database.

**Table 4:** PANTHER GO-Slim biological process analysis.

Reference List		Reference List	
*Rattus norvegicus*	Fold Enrichment	*Mus musculus*	Fold Enrichment
protein K48-linked ubiquitination (GO:0070936)	47.06	regulation of endocytosis (GO:0030100)	27.45
regulation of endocytosis (GO:0030100)	26.89	actin filament organization (GO:0007015)	7.97
actin filament organization (GO:0007015)	7.93	translational elongation (GO:0006414)	6.56
ubiquitin-dependent protein catabolic process (GO:0006511)	5.88	translation (GO:0006412)	6.56
modification-dependent protein catabolic process (GO:0019941)	5.67	ribonucleoprotein complex biogenesis (GO:0022613)	6.53
modification-dependent macromolecule catabolic process (GO:0043632)	5.58	peptide biosynthetic process (GO:0043043)	6.47
regulation of cellular component organization (GO:0051128)	4.76	cellular protein-containing complex assembly (GO:0034622)	5.19
cellular protein-containing complex assembly (GO:0034622)	4.57	regulation of cellular component organization (GO:0051128)	4.87
intracellular protein transport (GO:0006886)	4.29	protein-containing complex assembly (GO:0065003)	4.87
protein transport (GO:0015031)	4.14	intracellular transport (GO:0046907)	4.47
peptide transport (GO:0015833)	4.09	intracellular protein transport (GO:0006886)	4.36
intracellular transport (GO:0046907)	4.08	protein transport (GO:0015031)	4.19
establishment of protein localization (GO:0045184)	4.05	peptide transport (GO:0015833)	4.16
amide transport (GO:0042886)	4.01	establishment of protein localization (GO:0045184)	4.11
establishment of localization in cell (GO:0051649)	3.74	establishment of localization in cell (GO:0051649)	4.07
nitrogen compound transport (GO:0071705)	3.72	amide transport (GO:0042886)	4.06
cellular component assembly (GO:0022607)	3.55	nitrogen compound transport (GO:0071705)	3.77
cellular component biogenesis (GO:0044085)	3.43	cellular component biogenesis (GO:0044085)	3.77
protein localization (GO:0008104)	3.4	cellular component assembly (GO:0022607)	3.76
cellular localization (GO:0051641)	3.18	cellular localization (GO:0051641)	3.47
cellular component organization or biogenesis (GO:0071840)	2.34	protein localization (GO:0008104)	3.41
cellular component organization (GO:0016043)	2.3	cellular component organization or biogenesis (GO:0071840)	2.36
		cellular component organization (GO:0016043)	2.28

All results shown are valid for an overall FDR< 0.05, and a raw *p*-value < 0.05, as determined by Fisher’s exact test. Fold enrichment is the gene expression observed in the uploaded list over the expected list. If it is greater than 1, this indicates that the category is overrepresented in our experiment. Conversely, the category is underrepresented if it is less than one. GO: Gene ontology database.

**Table 5:** PANTHER GO-Slim Cellular Component analysis.

Reference List		Reference List	
*Rattus norvegicus*	Fold Enrichment	*Mus musculus*	Fold Enrichment
proteasome regulatory particle, lid subcomplex (GO:0008541)	71.71	proteasome regulatory particle, lid subcomplex (GO:0008541)	61.76
proteasome accessory complex (GO:0022624)	50.2	proteasome accessory complex (GO:0022624)	46.32
proteasome regulatory particle (GO:0005838)	50.2	proteasome regulatory particle (GO:0005838)	46.32
eukaryotic translation initiation factor 3 complex (GO:0005852)	33.47	cytosolic small ribosomal subunit (GO:0022627)	36.33
endocytic vesicle (GO:0030139)	24.29	eukaryotic translation initiation factor 3 complex (GO:0005852)	35.29
proteasome complex (GO:0000502)	18.82	small ribosomal subunit (GO:0015935)	29.65
early endosome (GO:0005769)	17.93	cytosolic ribosome (GO:0022626)	29.54
endopeptidase complex (GO:1905369)	17.11	cytoplasmic stress granule (GO:0010494)	27.45
peptidase complex (GO:1905368)	12.98	cytosolic large ribosomal subunit (GO:0022625)	26.01
small ribosomal subunit (GO:0015935)	10.32	endocytic vesicle (GO:0030139)	25.56
cytosolic small ribosomal subunit (GO:0022627)	10.2	ribosomal subunit (GO:0044391)	22.98
actin cytoskeleton (GO:0015629)	7.84	ribosome (GO:0005840)	21.18
cytosolic ribosome (GO:0022626)	7.67	proteasome complex (GO:0000502)	19.5
ribosomal subunit (GO:0044391)	7.38	large ribosomal subunit (GO:0015934)	18.76
ribosome (GO:0005840)	7.09	early endosome (GO:0005769)	17.97
cytosolic large ribosomal subunit (GO:0022625)	6.38	endopeptidase complex (GO:1905369)	17.65
large ribosomal subunit (GO:0015934)	5.75	peptidase complex (GO:1905368)	12.56
ribonucleoprotein complex (GO:1990904)	5.42	ribonucleoprotein complex (GO:1990904)	9.34
cytosol (GO:0005829)	4.17	actin cytoskeleton (GO:0015629)	7.84
cytoskeleton (GO:0005856)	3.39	cytosol (GO:0005829)	5.53
intracellular non-membrane-bounded organelle (GO:0043232)	2.68	cytoplasmic vesicle (GO:0031410)	3.69
non-membrane-bounded organelle (GO:0043228)	2.68	intracellular vesicle (GO:0097708)	3.67
protein-containing complex (GO:0032991)	2.54	cytoskeleton (GO:0005856)	3.38
cytoplasm (GO:0005737)	2.4	intracellular non-membrane-bounded organelle (GO:0043232)	2.95
intracellular organelle (GO:0043229)	1.91	non-membrane-bounded organelle (GO:0043228)	2.95
organelle (GO:0043226)	1.87	protein-containing complex (GO:0032991)	2.81
intracellular (GO:0005622)	1.86	cytoplasm (GO:0005737)	2.68
cellular_component (GO:0005575)	1.38	intracellular organelle (GO:0043229)	2
cellular anatomical entity (GO:0110165)	1.32	organelle (GO:0043226)	1.96
		intracellular (GO:0005622)	1.92
		cellular_component (GO:0005575)	1.38
		cellular anatomical entity (GO:0110165)	1.32

All results shown are valid for an overall FDR < 0.05, and a raw *p*-value < 0.05, as determined by Fisher’s exact test. Fold enrichment is the gene observed in the uploaded list over that in the expected list. If it is greater than 1, the category is overrepresented in our experiment. Conversely, the category is underrepresented if it is less than 1. GO: Gene ontology database.

## Data Availability

The data supporting the findings of this study are available from the corresponding author, Yin-Hwa Shih, upon reasonable request.

## Supplementary Materials

The following are available online at https://www.mdpi.com/article/10.3390/diagnostics12010065/s1, Table S1: 174 unique proteins absent in salivary exosomes from the PreD group. Figure S1: Detection of CD63 expression of salivary exosome proteins from 3 hamsters among PreD group. Supplementary File 1: 136 Reactome pathways in the Rattus norvegicus database and 132 Reactome pathways in the Mus musculus database.
