# Supplementary material for: Salivary Exosome Proteomics and Bioinformatics Analysis in 7,12-Dimethylbenz[a]anthracene-Induced Oral Cancer with Radiation Therapy—A Syrian Golden Hamster Model
Source: Diagnostics (Basel). 2021 Dec 28;12(1):65. doi: 10.3390/diagnostics12010065 (PMC8774811; doi:10.3390/diagnostics12010065)
Supplement: Supplementary file 1 [file diagnostics-12-00065-s001.zip › Supplementary Table S1.pdf]

Supplementary Table S1. 174 unique proteins absence in salivary exosomes in PreD group.

Protein name	Gene name
forkhead-associated domain-containing protein 1 OS=Mesocricetus auratus OX=10036 GN=Fhad1 PE=4 SV=1	Fhad1
V-type proton ATPase catalytic subunit A (Fragments) OS=Mesocricetus auratus OX=10036 GN=ATP6V1A PE=1 SV=1	ATP6V1A
40S ribosomal protein S4 OS=Mesocricetus auratus OX=10036 GN=Rps4x PE=3 SV=1	Rps4x
Septin OS=Mesocricetus auratus OX=10036 GN=Sept8 PE=3 SV=1	Sept8
Annexin OS=Mesocricetus auratus OX=10036 GN=Anxa7 PE=3 SV=1	Anxa7
polypyrimidine tract-binding protein 3 OS=Mesocricetus auratus OX=10036 GN=Ptbp3 PE=4 SV=2	Ptbp3
bromodomain-containing protein 4 isoform X3 OS=Mesocricetus auratus OX=10036 GN=Brd4 PE=4 SV=1	Brd4
spectrin alpha chain, non-erythrocytic 1 isoform X2 OS=Mesocricetus auratus OX=10036 GN=Sptan1 PE=3 SV=1	Sptan1
Eukaryotic translation initiation factor 5A OS=Mesocricetus auratus OX=10036 GN=Eif5al1 PE=3 SV=1	Eif5al1
Synaptogyrin OS=Mesocricetus auratus OX=10036 GN=Syngr2 PE=3 SV=1	Syngr2
tropomyosin alpha-1 chain isoform X16 OS=Mesocricetus auratus OX=10036 GN=Tpm1 PE=3 SV=1	Tpm1
tropomyosin alpha-1 chain isoform X10 OS=Mesocricetus auratus OX=10036 GN=Tpm1 PE=3 SV=1	Tpm1
Ubiquitinyl hydrolase 1 OS=Mesocricetus auratus OX=10036 GN=Usp9x PE=3 SV=1	Usp9x
tropomyosin beta chain isoform X2 OS=Mesocricetus auratus OX=10036 GN=Tpm2 PE=3 SV=1	Tpm2
bromodomain-containing protein 4 isoform X4 OS=Mesocricetus auratus OX=10036 GN=Brd4 PE=4 SV=1	Brd4
ras-related protein Rab-5A OS=Mesocricetus auratus OX=10036 GN=Rab5a PE=4 SV=1	Rab5a
60S ribosomal protein L13 OS=Mesocricetus auratus OX=10036 GN=Rpl13 PE=3 SV=2	Rpl13
H(+)-transporting two-sector ATPase OS=Mesocricetus auratus OX=10036 GN=Atp6v1a PE=3 SV=1	Atp6v1a
Actin-related protein 2/3 complex subunit 3 OS=Mesocricetus auratus OX=10036 GN=Arpc3 PE=3 SV=1	Arpc3
bromodomain-containing protein 4 isoform X1 OS=Mesocricetus auratus OX=10036 GN=Brd4 PE=4 SV=1	Brd4
CCT-beta OS=Mesocricetus auratus OX=10036 GN=Cct2 PE=3 SV=1	LOC101843624; Cct2

nascent polypeptide-associated complex subunit alpha OS=Mesocricetus auratus OX=10036 GN=LOC101829818 PE=4 SV=1	Naca; LOC101829818
coatomer subunit alpha-like OS=Mesocricetus auratus OX=10036 GN=LOC110339977 PE=4 SV=1	LOC110339977
spectrin alpha chain, non-erythrocytic 1 isoform X4 OS=Mesocricetus auratus OX=10036 GN=Sptan1 PE=3 SV=1	Sptan1
spectrin alpha chain, non-erythrocytic 1 isoform X5 OS=Mesocricetus auratus OX=10036 GN=Sptan1 PE=3 SV=1	Sptan1
plakophilin-3 isoform X2 OS=Mesocricetus auratus OX=10036 GN=Pkp3 PE=3 SV=1	Pkp3
Ubiquitin-activating enzyme E1 OS=Mesocricetus auratus OX=10036 GN=Uba1 PE=3 SV=1	Uba1
F-actin-capping protein subunit alpha OS=Mesocricetus auratus OX=10036 GN=Capza1 PE=3 SV=1	Capza1
sorcin isoform X2 OS=Mesocricetus auratus OX=10036 GN=Sri PE=4 SV=1	Sri
sorcin isoform X1 OS=Mesocricetus auratus OX=10036 GN=Sri PE=4 SV=1	Sri
Septin OS=Mesocricetus auratus OX=10036 GN=Sept6 PE=3 SV=1	Sept6
ubiquitin-conjugating enzyme E2 D2B OS=Mesocricetus auratus OX=10036 GN=LOC101826197 PE=3 SV=1	LOC101826197
cellular retinoic acid-binding protein 2 OS=Mesocricetus auratus OX=10036 GN=Crabp2 PE=3 SV=1	Crabp2
cAMP-dependent protein kinase type II-alpha regulatory subunit OS=Mesocricetus auratus OX=10036 GN=Prkar2a PE=3 SV=1	Prkar2a
spectrin alpha chain, non-erythrocytic 1 isoform X1 OS=Mesocricetus auratus OX=10036 GN=Sptan1 PE=3 SV=1	Sptan1
melanoma inhibitory activity protein 3 OS=Mesocricetus auratus OX=10036 GN=Mia3 PE=4 SV=1	Mia3
Thymidine phosphorylase OS=Mesocricetus auratus OX=10036 GN=Tymp PE=3 SV=1	Tymp
complement component C8 gamma chain isoform X1 OS=Mesocricetus auratus OX=10036 GN=C8g PE=4 SV=1	C8g
ras-related protein Rab-5B OS=Mesocricetus auratus OX=10036 GN=Rab5b PE=4 SV=1	Rab5b
transportin-1 OS=Mesocricetus auratus OX=10036 GN=Tnpo1 PE=4 SV=1	Tnpo1
40S ribosomal protein S18 OS=Mesocricetus auratus OX=10036 GN=Rps18 PE=3 SV=1	Rps18
tripartite motif-containing protein 29 isoform X2 OS=Mesocricetus auratus OX=10036 GN=Trim29 PE=4 SV=1	Trim29
kallikrein-15 OS=Mesocricetus auratus OX=10036 GN=Klk15 PE=4 SV=1	LOC101838896; Klk15
transmembrane emp24 domain-containing protein 9 OS=Mesocricetus auratus OX=10036 GN=Tmed9 PE=3 SV=1	Tmed9

poly(RC)-binding protein 3 isoform X4 OS=Mesocricetus auratus OX=10036 GN=Pcbp3 PE=4 SV=1	Pcbp3
importin subunit beta-1 OS=Mesocricetus auratus OX=10036 GN=Kpnb1 PE=3 SV=1	Kpnb1
Eukaryotic translation initiation factor 3 subunit A OS=Mesocricetus auratus OX=10036 GN=Eif3a PE=3 SV=1	Eif3a
tropomyosin alpha-1 chain isoform X5 OS=Mesocricetus auratus OX=10036 GN=Tpm1 PE=3 SV=1	Tpm1
Septin OS=Mesocricetus auratus OX=10036 GN=Sept6 PE=3 SV=1	Sept6
Ezrin (Fragments) OS=Mesocricetus auratus OX=10036 GN=EZR PE=1 SV=1	EZR
Septin OS=Mesocricetus auratus OX=10036 GN=Sept11 PE=3 SV=1	Sept11
60S ribosomal protein L4 OS=Mesocricetus auratus OX=10036 GN=Rpl4 PE=3 SV=1	Rpl4
Histidine--tRNA ligase, cytoplasmic OS=Mesocricetus auratus OX=10036 GN=HARS1 PE=2 SV=1	HARS1
Beta-glucuronidase OS=Mesocricetus auratus OX=10036 GN=Gusb PE=3 SV=1	Gusb
40S ribosomal protein S4 OS=Mesocricetus auratus OX=10036 GN=LOC101838157 PE=3 SV=1	LOC101838157
serpin B7 OS=Mesocricetus auratus OX=10036 GN=Serpinb7 PE=3 SV=1	Serpinb7
Eukaryotic translation initiation factor 3 subunit I OS=Mesocricetus auratus OX=10036 GN=Eif3i PE=3 SV=1	Eif3i
tropomyosin alpha-1 chain isoform X4 OS=Mesocricetus auratus OX=10036 GN=Tpm1 PE=3 SV=1	Tpm1
Septin OS=Mesocricetus auratus OX=10036 GN=Sept6 PE=3 SV=1	Sept6
14-3-3 protein theta OS=Mesocricetus auratus OX=10036 GN=Ywhaq PE=3 SV=1	Ywhaq
Septin OS=Mesocricetus auratus OX=10036 GN=Sept8 PE=3 SV=1	Sept8
tropomyosin alpha-1 chain isoform X1 OS=Mesocricetus auratus OX=10036 GN=Tpm1 PE=3 SV=1	Tpm1
40S ribosomal protein S16 OS=Mesocricetus auratus OX=10036 GN=Rps16 PE=3 SV=1	Rps16
26S proteasome non-ATPase regulatory subunit 2 OS=Mesocricetus auratus OX=10036 GN=Psmd2 PE=3 SV=1	Psmd2
ras-related protein Rab-2A OS=Mesocricetus auratus OX=10036 GN=Rab2a PE=4 SV=1	Rab2a
methylosome protein 50 OS=Mesocricetus auratus OX=10036 GN=Wdr77 PE=4 SV=1	Wdr77
plakophilin-3 isoform X1 OS=Mesocricetus auratus OX=10036 GN=Pkp3 PE=3 SV=1	Pkp3

Polyadenylate-binding protein OS=Mesocricetus auratus OX=10036 GN=Pabpc1 PE=3 SV=2	Pabpc1
poly(RC)-binding protein 3 isoform X3 OS=Mesocricetus auratus OX=10036 GN=Pcbp3 PE=4 SV=1	Pcbp3
tropomyosin alpha-1 chain isoform X9 OS=Mesocricetus auratus OX=10036 GN=Tpm1 PE=3 SV=1	Tpm1
Vomeronasal type-1 receptor OS=Mesocricetus auratus OX=10036 GN=LOC101834146 PE=3 SV=1	LOC101834146
tropomyosin alpha-1 chain isoform X18 OS=Mesocricetus auratus OX=10036 GN=Tpm1 PE=3 SV=1	Tpm1
Phosphoglycerate kinase OS=Mesocricetus auratus OX=10036 GN=LOC110340293 PE=3 SV=1	LOC110340293
Histidine ammonia-lyase OS=Mesocricetus auratus OX=10036 GN=Hal PE=3 SV=1	Hal
Sulfotransferase OS=Mesocricetus auratus OX=10036 GN=LOC101842832 PE=3 SV=1	LOC101842832
tropomyosin alpha-1 chain isoform X7 OS=Mesocricetus auratus OX=10036 GN=Tpm1 PE=3 SV=1	Tpm1
tropomyosin alpha-1 chain isoform X13 OS=Mesocricetus auratus OX=10036 GN=Tpm1 PE=3 SV=1	Tpm1
Septin OS=Mesocricetus auratus OX=10036 GN=Sept6 PE=3 SV=1	Sept6
60S ribosomal protein L35a OS=Mesocricetus auratus OX=10036 GN=LOC110340547 PE=3 SV=1	LOC110340547
Septin OS=Mesocricetus auratus OX=10036 GN=Sept6 PE=3 SV=1	Sept6
40S ribosomal protein S3 OS=Mesocricetus auratus OX=10036 GN=Rps3 PE=3 SV=1	Rps3
AP-1 complex subunit gamma OS=Mesocricetus auratus OX=10036 GN=Ap1g1 PE=3 SV=1	Ap1g1
spectrin alpha chain, non-erythrocytic 1 isoform X6 OS=Mesocricetus auratus OX=10036 GN=Sptan1 PE=3 SV=1	Sptan1
Heterogeneous nuclear ribonucleoprotein K OS=Mesocricetus auratus OX=10036 GN=Hnrnpk PE=4 SV=1	Hnrnpk
tropomyosin alpha-1 chain isoform X3 OS=Mesocricetus auratus OX=10036 GN=Tpm1 PE=3 SV=1	Tpm1
ubiquitin-conjugating enzyme E2 D2 isoform X1 OS=Mesocricetus auratus OX=10036 GN=Ube2d2 PE=3 SV=1	Ube2d2
poly(RC)-binding protein 3 isoform X5 OS=Mesocricetus auratus OX=10036 GN=Pcbp3 PE=4 SV=1	Pcbp3
poly(RC)-binding protein 3 isoform X8 OS=Mesocricetus auratus OX=10036 GN=Pcbp3 PE=4 SV=1	Pcbp3
Septin OS=Mesocricetus auratus OX=10036 GN=Sept8 PE=3 SV=1	Sept8
tripartite motif-containing protein 29 isoform X1 OS=Mesocricetus auratus OX=10036 GN=Trim29 PE=4 SV=1	Trim29

Ribosomal protein OS=Mesocricetus auratus OX=10036 GN=Rpl10a PE=3 SV=1	Rpl10a
tropomyosin alpha-1 chain isoform X19 OS=Mesocricetus auratus OX=10036 GN=Tpm1 PE=3 SV=1	Tpm1
tropomyosin alpha-1 chain isoform X8 OS=Mesocricetus auratus OX=10036 GN=Tpm1 PE=3 SV=1	Tpm1
Fascin OS=Mesocricetus auratus OX=10036 GN=Fscn1 PE=3 SV=1	Fscn1
tropomyosin alpha-1 chain isoform X17 OS=Mesocricetus auratus OX=10036 GN=Tpm1 PE=3 SV=1	Tpm1
Protein S100 OS=Mesocricetus auratus OX=10036 GN=S100a16 PE=3 SV=1	S100a16
Small nuclear ribonucleoprotein Sm D3 OS=Mesocricetus auratus OX=10036 GN=Snrpd3 PE=3 SV=1	Snrpd3
ADP-ribosylation factor GTPase-activating protein 3 isoform X2 OS=Mesocricetus auratus OX=10036 GN=Arfgap3 PE=4 SV=1	Arfgap3
2-deoxy-D-ribose 5-phosphate aldolase OS=Mesocricetus auratus OX=10036 GN=Dera PE=4 SV=1	Dera
Ubiquitin thioesterase OS=Mesocricetus auratus OX=10036 GN=Otub1 PE=3 SV=1	Otub1
mitochondrial glutamate carrier 2 OS=Mesocricetus auratus OX=10036 GN=Slc25a18 PE=3 SV=1	Slc25a18
F-actin-capping protein subunit beta (Fragments) OS=Mesocricetus auratus OX=10036 GN=CAPZB PE=1 SV=1	CAPZB
poly(RC)-binding protein 3 isoform X7 OS=Mesocricetus auratus OX=10036 GN=Pcbp3 PE=4 SV=1	Pcbp3
26S proteasome non-ATPase regulatory subunit 3 OS=Mesocricetus auratus OX=10036 GN=Psmd3 PE=3 SV=1	Psmd3
Transferrin receptor protein 1 OS=Mesocricetus auratus OX=10036 GN=Tfrc PE=3 SV=1	Tfrc
Ribosomal protein L15 OS=Mesocricetus auratus OX=10036 GN=LOC101838491 PE=3 SV=2	LOC101838491
tropomyosin alpha-1 chain isoform X2 OS=Mesocricetus auratus OX=10036 GN=Tpm1 PE=3 SV=1	Tpm1
ubiquitin-conjugating enzyme E2 D3 OS=Mesocricetus auratus OX=10036 GN=Ube2d3 PE=3 SV=1	Ube2d3
estradiol 17 beta-dehydrogenase 5-like isoform X1 OS=Mesocricetus auratus OX=10036 GN=LOC101843311 PE=3 SV=1	LOC101843311
60S ribosomal protein L35a OS=Mesocricetus auratus OX=10036 GN=Rpl35a PE=3 SV=1	Rpl35a
tropomyosin alpha-1 chain isoform X6 OS=Mesocricetus auratus OX=10036 GN=Tpm1 PE=3 SV=1	Tpm1
tropomyosin alpha-1 chain isoform X14 OS=Mesocricetus auratus OX=10036 GN=Tpm1 PE=3 SV=1	Tpm1
spectrin alpha chain, non-erythrocytic 1 isoform X3 OS=Mesocricetus auratus OX=10036 GN=Sptan1 PE=3 SV=1	Sptan1

F-actin-capping protein subunit beta OS=Mesocricetus auratus OX=10036 GN=Capzb PE=3 SV=1	Capzb
WD repeat-containing protein 91 OS=Mesocricetus auratus OX=10036 GN=Wdr91 PE=3 SV=1	Wdr91
Tropomyosin-1 OS=Mesocricetus auratus OX=10036 GN=Tpm2 PE=2 SV=1	Tpm2
polyadenylate-binding protein 5 OS=Mesocricetus auratus OX=10036 GN=Pabpc5 PE=4 SV=1	Pabpc5
tropomyosin alpha-1 chain isoform X15 OS=Mesocricetus auratus OX=10036 GN=Tpm1 PE=3 SV=1	Tpm1
poly(RC)-binding protein 3 isoform X6 OS=Mesocricetus auratus OX=10036 GN=Pcbp3 PE=4 SV=1	Pcbp3
Glucose-6-phosphate 1-dehydrogenase OS=Mesocricetus auratus OX=10036 GN=G6pd PE=3 SV=1	G6pd
Ubiquitinyl hydrolase 1 OS=Mesocricetus auratus OX=10036 GN=Usp9x PE=3 SV=1	Usp9x
3-hydroxyhexobarbital dehydrogenase 1/3-alpha, 17-beta-hydroxysteroid dehydrogenase OS=Mesocricetus auratus OX=10036 GN=LOC101843311 PE=2 SV=1	LOC101843311
Annexin OS=Mesocricetus auratus OX=10036 GN=Anxa7 PE=3 SV=1	Anxa7
tropomyosin alpha-1 chain isoform X12 OS=Mesocricetus auratus OX=10036 GN=Tpm1 PE=3 SV=1	Tpm1
transportin-2 OS=Mesocricetus auratus OX=10036 GN=Tnpo2 PE=4 SV=1	Tnpo2
Aldehyde dehydrogenase OS=Mesocricetus auratus OX=10036 GN=Aldh3a1 PE=3 SV=1	Aldh3a1
epiplakin OS=Mesocricetus auratus OX=10036 GN=LOC101826226 PE=3 SV=1	LOC101826226
poly(RC)-binding protein 2 OS=Mesocricetus auratus OX=10036 GN=Pcbp2 PE=4 SV=1	Pcbp2
leucine-rich repeat-containing protein 15 OS=Mesocricetus auratus OX=10036 GN=LOC101840888 PE=4 SV=1	LOC101840070; LOC101840888
Delta-aminolevulinic acid dehydratase OS=Mesocricetus auratus OX=10036 GN=Alad PE=3 SV=1	Alad
Solute carrier organic anion transporter family member OS=Mesocricetus auratus OX=10036 GN=LOC101831756 PE=3 SV=1	LOC101831756
phosphatidylethanolamine-binding protein 1 OS=Mesocricetus auratus OX=10036 GN=Pebp1 PE=3 SV=1	Pebp1
60S ribosomal protein L11 OS=Mesocricetus auratus OX=10036 GN=Rpl11 PE=3 SV=1	Rpl11
ADP-ribosylation factor GTPase-activating protein 3 isoform X1 OS=Mesocricetus auratus OX=10036 GN=Arfgap3 PE=4 SV=1	Arfgap3

filamin-A OS=Mesocricetus auratus OX=10036 GN=LOC101842052 PE=3 SV=2	LOC101842052
40S ribosomal protein S11 OS=Mesocricetus auratus OX=10036 GN=Rps11 PE=3 SV=1	Rps11
WD repeat-containing protein 91 OS=Mesocricetus auratus OX=10036 GN=Wdr91 PE=3 SV=1	Wdr91
D-dopachrome decarboxylase OS=Mesocricetus auratus OX=10036 GN=LOC101844189 PE=3 SV=1	LOC101844189
40S ribosomal protein S4 OS=Mesocricetus auratus OX=10036 GN=LOC101839291 PE=3 SV=1	LOC101839291
poly(RC)-binding protein 3 isoform X2 OS=Mesocricetus auratus OX=10036 GN=Pcbp3 PE=4 SV=1	Pcbp3
tropomyosin alpha-1 chain isoform X11 OS=Mesocricetus auratus OX=10036 GN=Tpm1 PE=3 SV=1	Tpm1
poly(RC)-binding protein 3 isoform X1 OS=Mesocricetus auratus OX=10036 GN=Pcbp3 PE=4 SV=1	Pcbp3
poly(RC)-binding protein 1 OS=Mesocricetus auratus OX=10036 GN=Pcbp1 PE=4 SV=1	Pcbp1
tropomyosin beta chain isoform X1 OS=Mesocricetus auratus OX=10036 GN=Tpm2 PE=3 SV=1	Tpm2
2-phospho-D-glycerate hydro-lyase OS=Mesocricetus auratus OX=10036 GN=Eno2 PE=3 SV=1	Eno2
septin-11 isoform X3 OS=Mesocricetus auratus OX=10036 GN=Sept11 PE=3 SV=1	Sept11
F-actin-capping protein subunit beta OS=Mesocricetus auratus OX=10036 GN=Capzb PE=3 SV=1	Capzb
Ubiquitinyl hydrolase 1 OS=Mesocricetus auratus OX=10036 GN=Usp9x PE=3 SV=1	Usp9x
complement component C8 gamma chain isoform X2 OS=Mesocricetus auratus OX=10036 GN=C8g PE=4 SV=1	C8g
syntaxin-3 isoform X2 OS=Mesocricetus auratus OX=10036 GN=Stx3 PE=3 SV=1	Stx3
ras-related protein Rab-5C OS=Mesocricetus auratus OX=10036 GN=Rab5c PE=4 SV=1	Rab5c
ubiquitin-conjugating enzyme E2 D2 isoform X2 OS=Mesocricetus auratus OX=10036 GN=Ube2d2 PE=3 SV=1	Ube2d2
tropomyosin beta chain isoform X3 OS=Mesocricetus auratus OX=10036 GN=Tpm2 PE=3 SV=1	Tpm2
Acyl-peptide hydrolase OS=Mesocricetus auratus OX=10036 GN=Apeh PE=4 SV=1	Apeh
26S proteasome non-ATPase regulatory subunit 11 OS=Mesocricetus auratus OX=10036 GN=Psmd11 PE=3 SV=2	Psmd11
Ubiquitinyl hydrolase 1 OS=Mesocricetus auratus OX=10036 GN=Usp9x PE=3 SV=1	Usp9x
60S ribosomal protein L5 isoform X1 OS=Mesocricetus auratus OX=10036 GN=Rpl5 PE=3 SV=1	Rpl5

14-3-3 protein beta/alpha OS=Mesocricetus auratus OX=10036 GN=Ywhab PE=3 SV=1	Ywhab
Septin OS=Mesocricetus auratus OX=10036 GN=Sept11 PE=3 SV=1	Sept11
Platelet-activating factor acetylhydrolase IB subunit alpha OS=Mesocricetus auratus OX=10036 GN=Pafah1b1 PE=3 SV=1	Pafah1b1
Peptidyl-prolyl cis-trans isomerase OS=Mesocricetus auratus OX=10036 GN=LOC101837947 PE=3 SV=1	LOC101837947
60S ribosomal protein L5 isoform X3 OS=Mesocricetus auratus OX=10036 GN=Rpl5 PE=3 SV=1	Rpl5
F-actin-capping protein subunit beta OS=Mesocricetus auratus OX=10036 GN=Capzb PE=3 SV=1	Capzb
Galectin OS=Mesocricetus auratus OX=10036 GN=Lgalsl PE=4 SV=1	Lgalsl
cAMP-dependent protein kinase type II-beta regulatory subunit OS=Mesocricetus auratus OX=10036 GN=Prkar2b PE=3 SV=1	Prkar2b
60S ribosomal protein L23 OS=Mesocricetus auratus OX=10036 GN=Rpl23 PE=3 SV=1	Rpl23
AP-1 complex subunit gamma OS=Mesocricetus auratus OX=10036 GN=Ap1g1 PE=3 SV=1	Ap1g1
60S ribosomal protein L11 OS=Mesocricetus auratus OX=10036 GN=LOC101827706 PE=3 SV=1	LOC101827706
Solute carrier organic anion transporter family member OS=Mesocricetus auratus OX=10036 GN=LOC101831756 PE=3 SV=1	LOC101831756
40S ribosomal protein S2 OS=Mesocricetus auratus OX=10036 GN=Rps2 PE=4 SV=1	Rps2
bromodomain-containing protein 4 isoform X2 OS=Mesocricetus auratus OX=10036 GN=Brd4 PE=4 SV=1	Brd4
protein-glutamine gamma-glutamyltransferase 6 OS=Mesocricetus auratus OX=10036 GN=Tgm6 PE=3 SV=1	Tgm6
eukaryotic translation initiation factor 4E OS=Mesocricetus auratus OX=10036 GN=LOC101836188 PE=3 SV=1	LOC101836188
CCT-theta OS=Mesocricetus auratus OX=10036 GN=Cct8 PE=3 SV=1	Cct8

Supplementary Table 2. 136 reactome pathways in Rattus norvegicus database and 132 reactome pathways in Mus musculus database

Analysis Type:	PANTHER Overrepresentation Test (Released 20210224)
Annotation Version and Release Date:	Reactome version 65 Released 2020-11-17
Analyzed List:	upload_1 (Rattus norvegicus)
Reference List:	Rattus norvegicus (all genes in database)
Test Type:	FISHER
Correction:	FDR

Reactome pathways	Rattus norvegicus - REFLIST (21586)	upload_1 (86)	upload_1 (expected)	upload_1 (over/under)	upload_1 (fold Enrichment)	upload_1 (raw P-value)	upload_1 (FDR)
CREB1 phosphorylation through the activation of Adenylate Cyclase (R-RNO-442720)	5	2	0.02	+	> 100	3.23E-04	1.20E-02
PKA activation in glucagon signalling (R-RNO-164378)	5	2	0.02	+	> 100	3.23E-04	1.18E-02
Glucagon signaling in metabolic regulation (R-RNO-163359)	7	2	0.03	+	71.71	5.50E-04	1.74E-02
Regulation of localization of FOXO transcription factors (R-RNO-9614399)	12	2	0.05	+	41.83	1.37E-03	2.93E-02
Activation of BAD and translocation to mitochondria (R-RNO-111447)	12	2	0.05	+	41.83	1.37E-03	2.89E-02
Chk1/Chk2(Cds1) mediated inactivation of Cyclin B:Cdk1 complex (R-RNO-75035)	12	2	0.05	+	41.83	1.37E-03	2.85E-02
Formation of the ternary complex, and subsequently, the 43S complex (R-RNO-72695)	51	8	0.2	+	39.37	8.29E-11	7.16E-09
Translation initiation complex formation (R-RNO-72649)	58	9	0.23	+	38.95	5.63E-12	5.78E-10
Activation of the mRNA upon binding of the cap-binding complex and eIFs, and subsequent binding to 43S (R-RNO-72662)	59	9	0.24	+	38.29	6.46E-12	6.24E-10
Pentose phosphate pathway (R-RNO-71336)	14	2	0.06	+	35.86	1.80E-03	3.36E-02
Ribosomal scanning and start codon recognition (R-RNO-72702)	58	8	0.23	+	34.62	2.10E-10	1.72E-08
Formation of a pool of free 40S subunits (R-RNO-72689)	112	15	0.45	+	33.62	2.16E-18	7.11E-16
DARPP-32 events (R-RNO-180024)	15	2	0.06	+	33.47	2.04E-03	3.48E-02

PKA activation (R-RNO-163615)	15	2	0.06	+	33.47	2.04E-03	3.45E-02
PKA-mediated phosphorylation of CREB (R-RNO-111931)	15	2	0.06	+	33.47	2.04E-03	3.41E-02
Nonsense Mediated Decay (NMD) independent of the Exon Junction Complex (EJC) (R-RNO-975956)	106	14	0.42	+	33.15	3.79E-17	8.90E-15
L13a-mediated translational silencing of Ceruloplasmin expression (R-RNO-156827)	122	16	0.49	+	32.92	1.98E-19	1.63E-16
SRP-dependent cotranslational protein targeting to membrane (R-RNO-1799339)	104	13	0.41	+	31.38	1.04E-15	1.43E-13
Post NMDA receptor activation events (R-RNO-438064)	16	2	0.06	+	31.38	2.29E-03	3.64E-02
Cap-dependent Translation Initiation (R-RNO-72737)	130	16	0.52	+	30.89	5.01E-19	2.74E-16
Eukaryotic Translation Initiation (R-RNO-72613)	130	16	0.52	+	30.89	5.01E-19	2.06E-16
GTP hydrolysis and joining of the 60S ribosomal subunit (R-RNO-72706)	123	15	0.49	+	30.61	7.81E-18	2.14E-15
Tristetraprolin (TTP, ZFP36) binds and destabilizes mRNA (R-RNO-450513)	17	2	0.07	+	29.53	2.55E-03	3.73E-02
Nonsense Mediated Decay (NMD) enhanced by the Exon Junction Complex (EJC) (R-RNO-975957)	125	14	0.5	+	28.11	3.13E-16	5.71E-14
Nonsense-Mediated Decay (NMD) (R-RNO-927802)	125	14	0.5	+	28.11	3.13E-16	5.14E-14
Activation of BH3-only proteins (R-RNO-114452)	18	2	0.07	+	27.89	2.82E-03	4.00E-02
Synthesis of active ubiquitin: roles of E1 and E2 enzymes (R-RNO-8866652)	29	3	0.12	+	25.97	2.75E-04	1.08E-02
FOXO-mediated transcription (R-RNO-9614085)	20	2	0.08	+	25.1	3.41E-03	4.38E-02
Downregulation of SMAD2/3:SMAD4 transcriptional activity (R-RNO-2173795)	20	2	0.08	+	25.1	3.41E-03	4.35E-02
Oxygen-dependent proline hydroxylation of Hypoxia-inducible Factor Alpha (R-RNO-1234176)	62	5	0.25	+	20.24	6.96E-06	4.40E-04
Cellular response to hypoxia (R-RNO-1234174)	66	5	0.26	+	19.02	9.27E-06	5.64E-04
AUF1 (hnRNP D0) binds and destabilizes mRNA (R-RNO-450408)	54	4	0.22	+	18.59	8.33E-05	4.02E-03
Regulation of mRNA stability by proteins that bind AU-rich elements (R-RNO-450531)	83	6	0.33	+	18.14	1.46E-06	9.59E-05

Translation (R-RNO-72766)	236	17	0.94	+	18.08	1.57E-16	3.23E-14
rRNA processing in the nucleus and cytosol (R-RNO-8868773)	189	13	0.75	+	17.26	1.30E-12	1.65E-10
rRNA processing (R-RNO-72312)	189	13	0.75	+	17.26	1.30E-12	1.53E-10
Major pathway of rRNA processing in the nucleolus and cytosol (R-RNO-6791226)	189	13	0.75	+	17.26	1.30E-12	1.43E-10
RAB geranylgeranylation (R-RNO-8873719)	59	4	0.24	+	17.02	1.15E-04	5.40E-03
Protein ubiquitination (R-RNO-8852135)	61	4	0.24	+	16.46	1.30E-04	5.77E-03
TP53 Regulates Metabolic Genes (R-RNO-5628897)	47	3	0.19	+	16.02	1.03E-03	2.78E-02
Cross-presentation of soluble exogenous antigens (endosomes) (R-RNO-1236978)	48	3	0.19	+	15.69	1.09E-03	2.77E-02
Ubiquitin-dependent degradation of Cyclin D (R-RNO-75815)	49	3	0.2	+	15.37	1.16E-03	2.88E-02
COPI-independent Golgi-to-ER retrograde traffic (R-RNO-6811436)	49	3	0.2	+	15.37	1.16E-03	2.84E-02
Regulation of RUNX2 expression and activity (R-RNO-8939902)	49	3	0.2	+	15.37	1.16E-03	2.80E-02
Regulation of ornithine decarboxylase (ODC) (R-RNO-350562)	50	3	0.2	+	15.06	1.22E-03	2.91E-02
p53-Independent G1/S DNA damage checkpoint (R-RNO-69613)	50	3	0.2	+	15.06	1.22E-03	2.87E-02
p53-Independent DNA Damage Response (R-RNO-69610)	50	3	0.2	+	15.06	1.22E-03	2.83E-02
Ubiquitin Mediated Degradation of Phosphorylated Cdc25A (R-RNO-69601)	50	3	0.2	+	15.06	1.22E-03	2.79E-02
Autodegradation of the E3 ubiquitin ligase COP1 (R-RNO-349425)	50	3	0.2	+	15.06	1.22E-03	2.75E-02
Regulation of RUNX3 expression and activity (R-RNO-8941858)	51	3	0.2	+	14.76	1.29E-03	2.83E-02
Degradation of AXIN (R-RNO-4641257)	52	3	0.21	+	14.48	1.36E-03	2.95E-02
FBXL7 down-regulates AURKA during mitotic entry and in early mitosis (R-RNO-8854050)	53	3	0.21	+	14.21	1.44E-03	2.95E-02
Degradation of GLI1 by the proteasome (R-RNO-5610780)	54	3	0.22	+	13.94	1.51E-03	3.07E-02
Stabilization of p53 (R-RNO-69541)	54	3	0.22	+	13.94	1.51E-03	3.03E-02
Degradation of DVL (R-RNO-4641258)	55	3	0.22	+	13.69	1.59E-03	3.15E-02

GLI3 is processed to GLI3R by the proteasome (R-RNO-5610785)	55	3	0.22	+	13.69	1.59E-03	3.11E-02
Dectin-1 mediated noncanonical NF-kB signaling (R-RNO-5607761)	56	3	0.22	+	13.45	1.67E-03	3.23E-02
NIK-->noncanonical NF-kB signaling (R-RNO-5676590)	56	3	0.22	+	13.45	1.67E-03	3.19E-02
CDT1 association with the CDC6:ORC:origin complex (R-RNO-68827)	57	3	0.23	+	13.21	1.75E-03	3.31E-02
COPI-mediated anterograde transport (R-RNO-6807878)	96	5	0.38	+	13.07	5.14E-05	2.81E-03
Metabolism of polyamines (R-RNO-351202)	58	3	0.23	+	12.98	1.84E-03	3.39E-02
Activation of NF-kappaB in B cells (R-RNO-1169091)	59	3	0.24	+	12.76	1.93E-03	3.48E-02
SCF(Skp2)-mediated degradation of p27/p21 (R-RNO-187577)	59	3	0.24	+	12.76	1.93E-03	3.44E-02
Asymmetric localization of PCP proteins (R-RNO-4608870)	60	3	0.24	+	12.55	2.02E-03	3.56E-02
Hedgehog ligand biogenesis (R-RNO-5358346)	60	3	0.24	+	12.55	2.02E-03	3.52E-02
Transcriptional regulation by RUNX2 (R-RNO-8878166)	60	3	0.24	+	12.55	2.02E-03	3.49E-02
Autodegradation of Cdh1 by Cdh1:APC/C (R-RNO-174084)	61	3	0.24	+	12.34	2.11E-03	3.47E-02
Hedgehog 'off' state (R-RNO-5610787)	105	5	0.42	+	11.95	7.72E-05	3.96E-03
p53-Dependent G1/S DNA damage checkpoint (R-RNO-69580)	63	3	0.25	+	11.95	2.31E-03	3.64E-02
p53-Dependent G1 DNA Damage Response (R-RNO-69563)	63	3	0.25	+	11.95	2.31E-03	3.60E-02
CLEC7A (Dectin-1) signaling (R-RNO-5607764)	85	4	0.34	+	11.81	4.36E-04	1.49E-02
Peroxisomal protein import (R-RNO-9033241)	64	3	0.25	+	11.77	2.41E-03	3.69E-02
Assembly of the pre-replicative complex (R-RNO-68867)	64	3	0.25	+	11.77	2.41E-03	3.66E-02
RUNX1 regulates transcription of genes involved in differentiation of HSCs (R-RNO-8939236)	65	3	0.26	+	11.58	2.51E-03	3.78E-02
G1/S DNA Damage Checkpoints (R-RNO-69615)	65	3	0.26	+	11.58	2.51E-03	3.75E-02
APC/C:Cdc20 mediated degradation of Securin (R-RNO-174154)	65	3	0.26	+	11.58	2.51E-03	3.71E-02
Regulation of RAS by GAPs (R-RNO-5658442)	66	3	0.26	+	11.41	2.62E-03	3.80E-02

Downstream TCR signaling (R-RNO-202424)	89	4	0.35	+	11.28	5.16E-04	1.69E-02
Regulation of PTEN stability and activity (R-RNO-8948751)	67	3	0.27	+	11.24	2.73E-03	3.89E-02
Orc1 removal from chromatin (R-RNO-68949)	68	3	0.27	+	11.07	2.84E-03	3.98E-02
Cyclin E associated events during G1/S transition (R-RNO-69202)	68	3	0.27	+	11.07	2.84E-03	3.95E-02
Factors involved in megakaryocyte development and platelet production (R-RNO-983231)	115	5	0.46	+	10.91	1.17E-04	5.32E-03
MAPK6/MAPK4 signaling (R-RNO-5687128)	70	3	0.28	+	10.76	3.07E-03	4.20E-02
CDK-mediated phosphorylation and removal of Cdc6 (R-RNO-69017)	70	3	0.28	+	10.76	3.07E-03	4.17E-02
Cyclin A:Cdk2-associated events at S phase entry (R-RNO-69656)	70	3	0.28	+	10.76	3.07E-03	4.13E-02
Cdc20:Phospho-APC/C mediated degradation of Cyclin A (R-RNO-174184)	70	3	0.28	+	10.76	3.07E-03	4.10E-02
APC/C:Cdh1 mediated degradation of Cdc20 and other APC/C:Cdh1 targeted proteins in late mitosis/early G1 (R-RNO-174178)	70	3	0.28	+	10.76	3.07E-03	4.07E-02
APC:Cdc20 mediated degradation of cell cycle proteins prior to satisfation of the cell cycle checkpoint (R-RNO-179419)	71	3	0.28	+	10.61	3.19E-03	4.19E-02
Downstream signaling events of B Cell Receptor (BCR) (R-RNO-1168372)	71	3	0.28	+	10.61	3.19E-03	4.16E-02
The role of GTSE1 in G2/M progression after G2 checkpoint (R-RNO-8852276)	72	3	0.29	+	10.46	3.32E-03	4.29E-02
ER to Golgi Anterograde Transport (R-RNO-199977)	146	6	0.58	+	10.32	3.19E-05	1.81E-03
APC/C:Cdc20 mediated degradation of mitotic proteins (R-RNO-176409)	73	3	0.29	+	10.32	3.44E-03	4.35E-02
UCH proteinases (R-RNO-5689603)	74	3	0.29	+	10.18	3.57E-03	4.48E-02
Activation of APC/C and APC/C:Cdc20 mediated degradation of mitotic proteins (R-RNO-176814)	74	3	0.29	+	10.18	3.57E-03	4.44E-02
Transcriptional regulation by RUNX3 (R-RNO-8878159)	74	3	0.29	+	10.18	3.57E-03	4.41E-02
Degradation of beta-catenin by the destruction complex (R-RNO-195253)	76	3	0.3	+	9.91	3.84E-03	4.70E-02
Golgi-to-ER retrograde transport (R-RNO-8856688)	127	5	0.51	+	9.88	1.82E-04	7.48E-03

Apoptosis (R-RNO-109581)	102	4	0.41	+	9.84	8.44E-04	2.39E-02
Metabolism of RNA (R-RNO-8953854)	563	22	2.24	+	9.81	7.06E-16	1.05E-13
Antigen processing-Cross presentation (R-RNO-1236975)	78	3	0.31	+	9.65	4.12E-03	4.94E-02
C-type lectin receptors (CLRs) (R-RNO-5621481)	104	4	0.41	+	9.65	9.05E-04	2.52E-02
TCR signaling (R-RNO-202403)	108	4	0.43	+	9.3	1.04E-03	2.74E-02
Programmed Cell Death (R-RNO-5357801)	108	4	0.43	+	9.3	1.04E-03	2.70E-02
Signaling by Hedgehog (R-RNO-5358351)	136	5	0.54	+	9.23	2.48E-04	9.92E-03
G2/M Checkpoints (R-RNO-69481)	143	5	0.57	+	8.78	3.10E-04	1.18E-02
Transport to the Golgi and subsequent modification (R-RNO-948021)	174	6	0.69	+	8.66	8.19E-05	4.08E-03
Ub-specific processing proteases (R-RNO-5689880)	158	5	0.63	+	7.94	4.82E-04	1.62E-02
FCERI mediated NF-kB activation (R-RNO-2871837)	131	4	0.52	+	7.66	2.06E-03	3.42E-02
Clathrin-mediated endocytosis (R-RNO-8856828)	141	4	0.56	+	7.12	2.67E-03	3.85E-02
G2/M Transition (R-RNO-69275)	177	5	0.71	+	7.09	7.95E-04	2.33E-02
Mitotic G2-G2/M phases (R-RNO-453274)	178	5	0.71	+	7.05	8.14E-04	2.35E-02
Intra-Golgi and retrograde Golgi-to-ER traffic (R-RNO-6811442)	186	5	0.74	+	6.75	9.86E-04	2.70E-02
RAF/MAP kinase cascade (R-RNO-5673001)	233	6	0.93	+	6.46	3.81E-04	1.36E-02
MAPK1/MAPK3 signaling (R-RNO-5684996)	239	6	0.95	+	6.3	4.34E-04	1.52E-02
Neutrophil degranulation (R-RNO-6798695)	489	12	1.95	+	6.16	6.62E-07	4.53E-05
FLT3 Signaling (R-RNO-9607240)	248	6	0.99	+	6.07	5.25E-04	1.69E-02
Asparagine N-linked glycosylation (R-RNO-446203)	257	6	1.02	+	5.86	6.30E-04	1.95E-02
Metabolism of proteins (R-RNO-392499)	1603	37	6.39	+	5.79	1.68E-19	2.76E-16
Cell Cycle Checkpoints (R-RNO-69620)	260	6	1.04	+	5.79	6.69E-04	2.03E-02
Neddylation (R-RNO-8951664)	217	5	0.86	+	5.78	1.92E-03	3.50E-02

MAPK family signaling cascades (R-RNO-5683057)	261	6	1.04	+	5.77	6.82E-04	2.04E-02
Deubiquitination (R-RNO-5688426)	225	5	0.9	+	5.58	2.23E-03	3.63E-02
Antigen processing: Ubiquitination & Proteasome degradation (R-RNO-983168)	286	6	1.14	+	5.27	1.08E-03	2.78E-02
Cellular responses to stress (R-RNO-2262752)	385	8	1.53	+	5.22	1.68E-04	7.28E-03
Cellular responses to external stimuli (R-RNO-8953897)	387	8	1.54	+	5.19	1.74E-04	7.34E-03
Membrane Trafficking (R-RNO-199991)	553	11	2.2	+	4.99	1.41E-05	8.26E-04
Intracellular signaling by second messengers (R-RNO-9006925)	257	5	1.02	+	4.88	3.91E-03	4.72E-02
Innate Immune System (R-RNO-168249)	938	17	3.74	+	4.55	1.75E-07	1.31E-05
Class I MHC mediated antigen processing & presentation (R-RNO-983169)	332	6	1.32	+	4.54	2.27E-03	3.66E-02
M Phase (R-RNO-68886)	333	6	1.33	+	4.52	2.31E-03	3.57E-02
Vesicle-mediated transport (R-RNO-5653656)	642	11	2.56	+	4.3	5.38E-05	2.85E-03
Post-translational protein modification (R-RNO-597592)	1218	20	4.85	+	4.12	5.79E-08	4.53E-06
Cell Cycle (R-RNO-1640170)	525	8	2.09	+	3.82	1.26E-03	2.79E-02
Immune System (R-RNO-168256)	1739	23	6.93	+	3.32	2.33E-07	1.66E-05
Adaptive Immune System (R-RNO-1280218)	745	9	2.97	+	3.03	3.00E-03	4.14E-02
Metabolism (R-RNO-1430728)	1713	15	6.82	+	2.2	3.89E-03	4.73E-02

Analysis Type:	PANTHER Overrepresentation Test (Released 20210224)
Annotation Version and Release Date:	Reactome version 65 Released 2020-11-17
Analyzed List:	upload_1 (Mus musculus)
Reference List:	Mus musculus (all genes in database)
Test Type:	FISHER
Correction:	FDR

Reactome pathways	Mus musculus - REFLIST (21988)	upload_1 (89)	upload_1 (expected)	upload_1 (over/under)	upload_1 (fold Enrichment)	upload_1 (raw P-value)	upload_1 (FDR)
CREB1 phosphorylation through the activation of Adenylate Cyclase (R-MMU-442720)	5	2	0.02	+	98.82	3.33E-04	1.25E-02
PKA activation in glucagon signalling (R-MMU-164378)	5	2	0.02	+	98.82	3.33E-04	1.22E-02
Glucagon signaling in metabolic regulation (R-MMU-163359)	7	2	0.03	+	70.59	5.68E-04	1.87E-02
Regulation of localization of FOXO transcription factors (R-MMU-9614399)	12	2	0.05	+	41.18	1.42E-03	3.12E-02
Activation of BAD and translocation to mitochondria (R-MMU-111447)	12	2	0.05	+	41.18	1.42E-03	3.07E-02
Chk1/Chk2(Cds1) mediated inactivation of Cyclin B:Cdk1 complex (R-MMU-75035)	12	2	0.05	+	41.18	1.42E-03	3.03E-02
Nonsense Mediated Decay (NMD) independent of the Exon Junction Complex (EJC) (R-MMU-975956)	91	14	0.37	+	38.01	7.02E-18	1.45E-15
Formation of the ternary complex, and subsequently, the 43S complex (R-MMU-72695)	52	8	0.21	+	38.01	1.09E-10	9.47E-09
Formation of a pool of free 40S subunits (R-MMU-72689)	98	15	0.4	+	37.81	4.62E-19	1.90E-16
Translation initiation complex formation (R-MMU-72649)	59	9	0.24	+	37.69	7.54E-12	7.31E-10
Activation of the mRNA upon binding of the cap-binding complex and eIFs, and subsequent binding to 43S (R-MMU-72662)	60	9	0.24	+	37.06	8.64E-12	7.91E-10
L13a-mediated translational silencing of Ceruloplasmin expression (R-MMU-156827)	108	16	0.44	+	36.6	4.52E-20	7.46E-17
SRP-dependent cotranslational protein targeting to membrane (R-MMU-1799339)	89	13	0.36	+	36.09	2.09E-16	2.87E-14

Cap-dependent Translation Initiation (R-MMU-72737)	116	16	0.47	+	34.08	1.28E-19	1.05E-16
Eukaryotic Translation Initiation (R-MMU-72613)	116	16	0.47	+	34.08	1.28E-19	7.03E-17
GTP hydrolysis and joining of the 60S ribosomal subunit (R-MMU-72706)	109	15	0.44	+	34	1.97E-18	5.40E-16
Ribosomal scanning and start codon recognition (R-MMU-72702)	59	8	0.24	+	33.5	2.72E-10	2.24E-08
DARPP-32 events (R-MMU-180024)	15	2	0.06	+	32.94	2.10E-03	3.69E-02
Pentose phosphate pathway (R-MMU-71336)	15	2	0.06	+	32.94	2.10E-03	3.65E-02
PKA activation (R-MMU-163615)	15	2	0.06	+	32.94	2.10E-03	3.61E-02
PKA-mediated phosphorylation of CREB (R-MMU-111931)	15	2	0.06	+	32.94	2.10E-03	3.57E-02
Nonsense Mediated Decay (NMD) enhanced by the Exon Junction Complex (EJC) (R-MMU-975957)	112	14	0.45	+	30.88	9.87E-17	1.63E-14
Nonsense-Mediated Decay (NMD) (R-MMU-927802)	112	14	0.45	+	30.88	9.87E-17	1.48E-14
Post NMDA receptor activation events (R-MMU-438064)	16	2	0.06	+	30.88	2.36E-03	3.77E-02
Tristetraprolin (TTP, ZFP36) binds and destabilizes mRNA (R-MMU-450513)	17	2	0.07	+	29.07	2.63E-03	3.98E-02
Activation of BH3-only proteins (R-MMU-114452)	18	2	0.07	+	27.45	2.91E-03	4.21E-02
IKK complex recruitment mediated by RIP1 (R-MMU-937041)	20	2	0.08	+	24.71	3.52E-03	4.61E-02
Synthesis of active ubiquitin: roles of E1 and E2 enzymes (R-MMU-8866652)	30	3	0.12	+	24.71	3.17E-04	1.24E-02
Oxygen-dependent proline hydroxylation of Hypoxia-inducible Factor Alpha (R-MMU-1234176)	65	5	0.26	+	19	9.36E-06	5.51E-04
Translation (R-MMU-72766)	224	17	0.91	+	18.75	9.49E-17	1.74E-14
Major pathway of rRNA processing in the nucleolus and cytosol (R-MMU-6791226)	174	13	0.7	+	18.46	6.09E-13	7.72E-11
rRNA processing in the nucleus and cytosol (R-MMU-8868773)	174	13	0.7	+	18.46	6.09E-13	7.17E-11
rRNA processing (R-MMU-72312)	174	13	0.7	+	18.46	6.09E-13	6.69E-11
AUF1 (hnRNP D0) binds and destabilizes mRNA (R-MMU-450408)	55	4	0.22	+	17.97	9.48E-05	4.74E-03

Cellular response to hypoxia (R-MMU-1234174)	70	5	0.28	+	17.65	1.31E-05	7.47E-04
Regulation of mRNA stability by proteins that bind AU-rich elements (R-MMU-450531)	85	6	0.34	+	17.44	1.83E-06	1.21E-04
RAB geranylgeranylation (R-MMU-8873719)	62	4	0.25	+	15.94	1.47E-04	6.73E-03
Cross-presentation of soluble exogenous antigens (endosomes) (R-MMU-1236978)	48	3	0.19	+	15.44	1.15E-03	3.00E-02
Protein ubiquitination (R-MMU-8852135)	64	4	0.26	+	15.44	1.65E-04	7.36E-03
TP53 Regulates Metabolic Genes (R-MMU-5628897)	49	3	0.2	+	15.13	1.21E-03	3.08E-02
COPI-independent Golgi-to-ER retrograde traffic (R-MMU-6811436)	49	3	0.2	+	15.13	1.21E-03	3.03E-02
Ubiquitin-dependent degradation of Cyclin D (R-MMU-75815)	50	3	0.2	+	14.82	1.28E-03	3.16E-02
Regulation of RUNX2 expression and activity (R-MMU-8939902)	50	3	0.2	+	14.82	1.28E-03	3.11E-02
Regulation of ornithine decarboxylase (ODC) (R-MMU-350562)	50	3	0.2	+	14.82	1.28E-03	3.06E-02
Autodegradation of the E3 ubiquitin ligase COP1 (R-MMU-349425)	51	3	0.21	+	14.53	1.35E-03	3.14E-02
p53-Independent G1/S DNA damage checkpoint (R-MMU-69613)	51	3	0.21	+	14.53	1.35E-03	3.10E-02
p53-Independent DNA Damage Response (R-MMU-69610)	51	3	0.21	+	14.53	1.35E-03	3.06E-02
Ubiquitin Mediated Degradation of Phosphorylated Cdc25A (R-MMU-69601)	51	3	0.21	+	14.53	1.35E-03	3.02E-02
Regulation of RUNX3 expression and activity (R-MMU-8941858)	53	3	0.21	+	13.98	1.50E-03	3.10E-02
Degradation of AXIN (R-MMU-4641257)	54	3	0.22	+	13.73	1.58E-03	3.22E-02
FBXL7 down-regulates AURKA during mitotic entry and in early mitosis (R-MMU-8854050)	54	3	0.22	+	13.73	1.58E-03	3.18E-02
Degradation of GLI1 by the proteasome (R-MMU-5610780)	55	3	0.22	+	13.48	1.66E-03	3.31E-02
Stabilization of p53 (R-MMU-69541)	55	3	0.22	+	13.48	1.66E-03	3.27E-02
Golgi Associated Vesicle Biogenesis (R-MMU-432722)	55	3	0.22	+	13.48	1.66E-03	3.23E-02
GLI3 is processed to GLI3R by the proteasome (R-MMU-5610785)	56	3	0.23	+	13.24	1.75E-03	3.32E-02
Degradation of DVL (R-MMU-4641258)	56	3	0.23	+	13.24	1.75E-03	3.28E-02

NIK-->noncanonical NF-kB signaling (R-MMU-5676590)	57	3	0.23	+	13	1.84E-03	3.40E-02
Dectin-1 mediated noncanonical NF-kB signaling (R-MMU-5607761)	57	3	0.23	+	13	1.84E-03	3.36E-02
COPI-mediated anterograde transport (R-MMU-6807878)	96	5	0.39	+	12.87	5.55E-05	2.95E-03
Metabolism of polyamines (R-MMU-351202)	58	3	0.23	+	12.78	1.93E-03	3.45E-02
CDT1 association with the CDC6:ORC:origin complex (R-MMU-68827)	58	3	0.23	+	12.78	1.93E-03	3.41E-02
SCF(Skp2)-mediated degradation of p27/p21 (R-MMU-187577)	60	3	0.24	+	12.35	2.11E-03	3.55E-02
Transcriptional regulation by RUNX2 (R-MMU-8878166)	61	3	0.25	+	12.15	2.21E-03	3.68E-02
Asymmetric localization of PCP proteins (R-MMU-4608870)	61	3	0.25	+	12.15	2.21E-03	3.64E-02
Autodegradation of Cdh1 by Cdh1:APC/C (R-MMU-174084)	62	3	0.25	+	11.95	2.31E-03	3.77E-02
Hedgehog ligand biogenesis (R-MMU-5358346)	62	3	0.25	+	11.95	2.31E-03	3.73E-02
Hedgehog 'off' state (R-MMU-5610787)	106	5	0.43	+	11.65	8.70E-05	4.49E-03
p53-Dependent G1/S DNA damage checkpoint (R-MMU-69580)	64	3	0.26	+	11.58	2.52E-03	3.95E-02
p53-Dependent G1 DNA Damage Response (R-MMU-69563)	64	3	0.26	+	11.58	2.52E-03	3.92E-02
Activation of NF-kappaB in B cells (R-MMU-1169091)	64	3	0.26	+	11.58	2.52E-03	3.88E-02
Peroxisomal protein import (R-MMU-9033241)	65	3	0.26	+	11.4	2.63E-03	4.01E-02
CLEC7A (Dectin-1) signaling (R-MMU-5607764)	87	4	0.35	+	11.36	5.05E-04	1.73E-02
APC/C:Cdc20 mediated degradation of Securin (R-MMU-174154)	66	3	0.27	+	11.23	2.74E-03	4.10E-02
G1/S DNA Damage Checkpoints (R-MMU-69615)	66	3	0.27	+	11.23	2.74E-03	4.07E-02
RUNX1 regulates transcription of genes involved in differentiation of HSCs (R-MMU-8939236)	66	3	0.27	+	11.23	2.74E-03	4.03E-02
Assembly of the pre-replicative complex (R-MMU-68867)	67	3	0.27	+	11.06	2.85E-03	4.16E-02
Regulation of PTEN stability and activity (R-MMU-8948751)	68	3	0.28	+	10.9	2.97E-03	4.26E-02
Regulation of RAS by GAPs (R-MMU-5658442)	68	3	0.28	+	10.9	2.97E-03	4.22E-02

Cyclin E associated events during G1/S transition (R-MMU-69202)	70	3	0.28	+	10.59	3.21E-03	4.53E-02
Orc1 removal from chromatin (R-MMU-68949)	70	3	0.28	+	10.59	3.21E-03	4.49E-02
Metabolism of RNA (R-MMU-8953854)	564	24	2.28	+	10.51	6.55E-18	1.54E-15
trans-Golgi Network Vesicle Budding (R-MMU-199992)	71	3	0.29	+	10.44	3.34E-03	4.59E-02
CDK-mediated phosphorylation and removal of Cdc6 (R-MMU-69017)	71	3	0.29	+	10.44	3.34E-03	4.55E-02
Cdc20:Phospho-APC/C mediated degradation of Cyclin A (R-MMU-174184)	71	3	0.29	+	10.44	3.34E-03	4.51E-02
APC/C:Cdh1 mediated degradation of Cdc20 and other APC/C:Cdh1 targeted proteins in late mitosis/early G1 (R-MMU-174178)	71	3	0.29	+	10.44	3.34E-03	4.48E-02
Downstream TCR signaling (R-MMU-202424)	95	4	0.38	+	10.4	6.94E-04	2.20E-02
Factors involved in megakaryocyte development and platelet production (R-MMU-983231)	119	5	0.48	+	10.38	1.47E-04	6.92E-03
APC:Cdc20 mediated degradation of cell cycle proteins prior to satisfation of the cell cycle checkpoint (R-MMU-179419)	72	3	0.29	+	10.29	3.47E-03	4.61E-02
Cyclin A:Cdk2-associated events at S phase entry (R-MMU-69656)	72	3	0.29	+	10.29	3.47E-03	4.58E-02
MAPK6/MAPK4 signaling (R-MMU-5687128)	73	3	0.3	+	10.15	3.60E-03	4.67E-02
The role of GTSE1 in G2/M progression after G2 checkpoint (R-MMU-8852276)	73	3	0.3	+	10.15	3.60E-03	4.64E-02
ER to Golgi Anterograde Transport (R-MMU-199977)	148	6	0.6	+	10.02	3.77E-05	2.07E-03
APC/C:Cdc20 mediated degradation of mitotic proteins (R-MMU-176409)	74	3	0.3	+	10.02	3.74E-03	4.70E-02
Activation of APC/C and APC/C:Cdc20 mediated degradation of mitotic proteins (R-MMU-176814)	75	3	0.3	+	9.88	3.87E-03	4.84E-02
Downstream signaling events of B Cell Receptor (BCR) (R-MMU-1168372)	76	3	0.31	+	9.75	4.02E-03	4.98E-02
Apoptosis (R-MMU-109581)	104	4	0.42	+	9.5	9.61E-04	2.83E-02
Golgi-to-ER retrograde transport (R-MMU-8856688)	130	5	0.53	+	9.5	2.18E-04	9.47E-03
C-type lectin receptors (CLRs) (R-MMU-5621481)	107	4	0.43	+	9.24	1.06E-03	2.92E-02

Signaling by Hedgehog (R-MMU-5358351)	138	5	0.56	+	8.95	2.85E-04	1.21E-02
Programmed Cell Death (R-MMU-5357801)	111	4	0.45	+	8.9	1.21E-03	3.13E-02
Clathrin-mediated endocytosis (R-MMU-8856828)	142	5	0.57	+	8.7	3.24E-04	1.24E-02
TCR signaling (R-MMU-202403)	114	4	0.46	+	8.67	1.33E-03	3.14E-02
G2/M Checkpoints (R-MMU-69481)	146	5	0.59	+	8.46	3.66E-04	1.31E-02
FCERI mediated NF-kB activation (R-MMU-2871837)	117	4	0.47	+	8.45	1.46E-03	3.06E-02
Transport to the Golgi and subsequent modification (R-MMU-948021)	178	6	0.72	+	8.33	1.01E-04	4.91E-03
Ub-specific processing proteases (R-MMU-5689880)	182	5	0.74	+	6.79	9.65E-04	2.79E-02
G2/M Transition (R-MMU-69275)	182	5	0.74	+	6.79	9.65E-04	2.74E-02
Mitotic G2-G2/M phases (R-MMU-453274)	184	5	0.74	+	6.71	1.01E-03	2.83E-02
Intra-Golgi and retrograde Golgi-to-ER traffic (R-MMU-6811442)	188	5	0.76	+	6.57	1.11E-03	2.96E-02
RAF/MAP kinase cascade (R-MMU-5673001)	239	6	0.97	+	6.2	4.73E-04	1.66E-02
MAPK1/MAPK3 signaling (R-MMU-5684996)	245	6	0.99	+	6.05	5.38E-04	1.81E-02
FLT3 Signaling (R-MMU-9607240)	254	6	1.03	+	5.84	6.47E-04	2.09E-02
Membrane Trafficking (R-MMU-199991)	562	13	2.27	+	5.71	5.10E-07	3.50E-05
Asparagine N-linked glycosylation (R-MMU-446203)	264	6	1.07	+	5.61	7.87E-04	2.45E-02
Metabolism of proteins (R-MMU-392499)	1639	37	6.63	+	5.58	7.71E-19	2.54E-16
Cell Cycle Checkpoints (R-MMU-69620)	266	6	1.08	+	5.57	8.18E-04	2.50E-02
MAPK family signaling cascades (R-MMU-5683057)	269	6	1.09	+	5.51	8.66E-04	2.60E-02
Neddylation (R-MMU-8951664)	225	5	0.91	+	5.49	2.40E-03	3.80E-02
Neutrophil degranulation (R-MMU-6798695)	547	12	2.21	+	5.42	2.49E-06	1.52E-04
Vesicle-mediated transport (R-MMU-5653656)	638	13	2.58	+	5.03	2.03E-06	1.29E-04
Antigen processing: Ubiquitination & Proteasome degradation (R-MMU-983168)	297	6	1.2	+	4.99	1.42E-03	3.01E-02

Deubiquitination (R-MMU-5688426)	250	5	1.01	+	4.94	3.73E-03	4.73E-02
Cellular responses to stress (R-MMU-2262752)	410	8	1.66	+	4.82	2.86E-04	1.18E-02
Cellular responses to external stimuli (R-MMU-8953897)	412	8	1.67	+	4.8	2.96E-04	1.19E-02
Innate Immune System (R-MMU-168249)	1013	18	4.1	+	4.39	1.26E-07	9.90E-06
M Phase (R-MMU-68886)	353	6	1.43	+	4.2	3.32E-03	4.60E-02
Class I MHC mediated antigen processing & presentation (R-MMU-983169)	361	6	1.46	+	4.11	3.70E-03	4.73E-02
Post-translational protein modification (R-MMU-597592)	1279	20	5.18	+	3.86	1.72E-07	1.29E-05
Cell Cycle (R-MMU-1640170)	550	8	2.23	+	3.59	1.86E-03	3.37E-02
Transport of small molecules (R-MMU-382551)	673	9	2.72	+	3.3	1.71E-03	3.28E-02
Adaptive Immune System (R-MMU-1280218)	760	10	3.08	+	3.25	1.06E-03	2.88E-02
Immune System (R-MMU-168256)	1830	24	7.41	+	3.24	1.97E-07	1.41E-05
