# Supplementary figures and images for: Salivary Exosome Proteomics and Bioinformatics Analysis in 7,12-Dimethylbenz[a]anthracene-Induced Oral Cancer with Radiation Therapy—A Syrian Golden Hamster Model
Source: Diagnostics (Basel). 2021 Dec 28;12(1):65. doi: 10.3390/diagnostics12010065 (PMC8774811; doi:10.3390/diagnostics12010065)

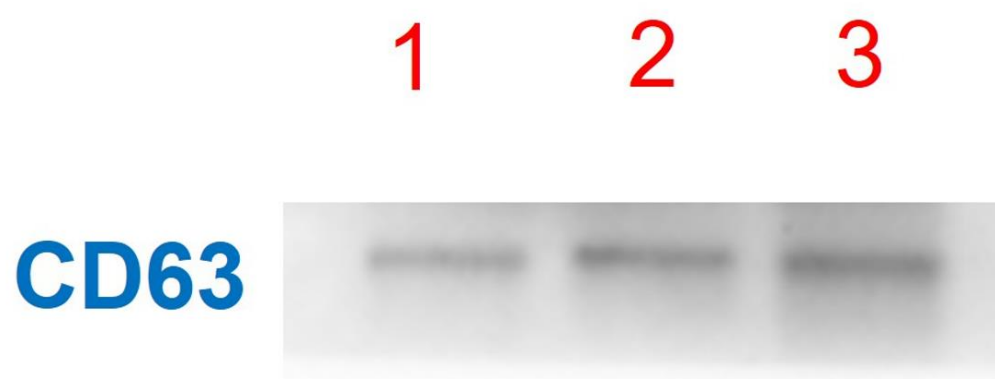

**Figure S1.** Detection of CD63 expression of salivary exosome proteins from 3 mice among PreD group.

Supplement: Supplementary file 1 [file diagnostics-12-00065-s001.zip › supplementary Figure S1.pdf]
